# Putative Novel Viruses in the Families *Lispiviridae* and *Rhabdoviridae* Detected in *Culex* and *Anopheles* Mosquitoes Collected at the São Paulo Zoo

**DOI:** 10.1155/av/8104754

**Published:** 2026-06-29

**Authors:** Lilian de Oliveira Guimarães, Roseane da Silva Couto, Geovani de Oliveira Ribeiro, Juliana Telles-de-Deus, Endrya do Socorro Foro Ramos, Vanessa Christe Helfstein, Vanessa dos Santos Morais, Jesus Maia dos Santos, Ramendra Pati Pandey, Vera Lucia Fonseca de Camargo-Neves, Antonio Charlys da Costa, Karin Kirchgatter, Élcio Leal

**Affiliations:** ^1^ Vector Section, Pasteur Institute, São Paulo, São Paulo, Brazil, saude.sp.gov.br; ^2^ Viral Diversity Laboratory, Institute of Biological Sciences, Federal University of Pará, Belém, Pará, Brazil, ufpa.br; ^3^ Leônidas and Maria Deane Institute (ILMD), Fiocruz Amazônia, Oswaldo Cruz Foundation, Manaus, Brazil, fiocruz.br; ^4^ Faculty of Medicine, Institute of Tropical Medicine, University of São Paulo, São Paulo, São Paulo, Brazil, usp.br; ^5^ Institute of Biosciences and Bioengineering, D. Y. Patil International University, Pune, Maharashtra, India

**Keywords:** *Anopheles*, *Culex*, Culicidae, metatranscriptomic, mosquitoes, virome

## Abstract

Several viruses belonging to the order *Mononegavirales* are recognized as highly pathogenic in humans and are often associated with lethal disease outcomes. Prominent examples include human respiratory syncytial virus (HRSV), Ebola virus (EBOV), rabies virus (RABV), and Marburg virus (MARV). In addition to their clinical severity, these viruses are considered potential biological threats due to their high transmissibility and virulence. In this study, we performed a metatranscriptomic analysis of *Culex* and *Anopheles* mosquitoes collected at the DBB (Diretoria de Biodiversidade e Biotecnologia), São Paulo, Brazil. Our analysis revealed viral sequences associated with two families within *Mononegavirales*: *Lispiviridae* and *Rhabdoviridae*. Phylogenetic analysis of the *Lispiviridae* sequences identified six variants, designated *CxLispV-SP_03*, *CxLispV-SP_09*, *CxLispV-SP_12*, *CxLispV-SP_13*, *CxLispV-SP_14*, and *CxLispV-SP_15*, that formed a strongly supported monophyletic clade with Canya virus, suggesting the discovery of a putative novel viral lineage. Examination of RNA‐dependent RNA polymerase (RdRp) domains in these sequences confirmed the presence of essential catalytic motifs. In contrast, the detected rhabdoviruses exhibited greater genomic structural heterogeneity, including the presence of accessory protein domains. Pairwise comparisons based on RdRp amino acid identity delineated four distinct groups among these sequences, pointing to substantial evolutionary divergence within the sampled *rhabdovirus* diversity. Together, these findings contribute to the growing characterization of mosquito‐associated mononegaviruses and provide a genomic foundation to support future surveillance efforts and public health risk assessments related to vector‐borne viruses.

## 1. Introduction

Mosquito‐based virological surveillance has become a strategic tool for investigating the diversity and dynamics of arthropod‐associated viruses. Although traditionally focused on the detection of medically important arboviruses, such as yellow fever, dengue, Zika, and chikungunya viruses, the incorporation of metagenomic approaches has revealed that the virome of these insects is substantially broader, encompassing a wide diversity of insect‐specific viruses (ISVs), many of which remain poorly characterized [1–3].

Among the RNA viruses detected in arthropods, the order *Mononegavirales* comprises negative‐sense single‐stranded RNA (−ssRNA) viruses exhibiting extensive genetic and organizational diversity. Within this order, well‐established families such as *Rhabdoviridae* coexist with more recently defined groups, including *Lispiviridae*. The latter was proposed to accommodate phylogenetically distinct lineages primarily identified through arthropod metagenomic studies, with genomes ranging from approximately 6.5 to 15.5 kb and genomic organization that is still undergoing taxonomic refinement [4].

Currently, the family *Lispiviridae* comprises 30 recognized genera and 45 species, with records spanning the Americas, Europe, Africa, Asia, and Oceania. Nevertheless, its global diversity remains underestimated, particularly in the Neotropical region, where systematic metagenomic surveys are still limited [4]. The incorporation of new complete genomes is therefore essential to strengthen phylogenetic inferences, refine taxonomic boundaries, and better understand evolutionary patterns within Mononegavirales.

In Brazil, the interface between preserved biomes and anthropized environments creates favorable scenarios for viral circulation in hematophagous arthropods, increasing the likelihood of detecting previously undescribed lineages [5]. Species occupying distinct ecological niches, such as members of the genera *Culex* (e.g., *Cx. chidesteri* and *Cx. renatoi*) and *Anopheles* (e.g., *An. strodei*), contribute to the maintenance of diverse viral communities in wild–urban transition zones [6]. Furthermore, recent evidence suggests that ISVs may modulate vector competence through immunological or competitive interactions, indirectly influencing pathogen transmission dynamics [7].

Despite the growing recognition of lispiviruses across different ecosystems and hosts, including insects of agricultural and sanitary importance, substantial gaps remain regarding their representation in Neotropical mosquitoes and their evolutionary placement relative to other *Mononegavirales* lineages. The characterization of novel genomes from Brazilian mosquitoes is therefore crucial to expanding current knowledge on the diversity, genomic organization, and evolutionary history of this viral family.

In this context, the present study describes the metagenomic analysis of mosquitoes of the genus *Culex* collected at the DBB (Diretoria de Biodiversidade e Biotecnologia), in the municipality of São Paulo, Brazil. Phylogenetic analysis of the RdRp coding region enabled the identification and classification of viruses belonging to the family *Lispiviridae,* closely related to the genus *Canmovirus*, as well as viruses within the family *Rhabdoviridae*, subfamilies *Alpharhabdovirinae* (*Ohlsrhavirus* and *Merhavirus*) and *Deltarhabdovirinae* (*Stangrhavirus*). In total, 15 viral genomes were characterized. The integration of robust phylogenetic inference, conserved protein domain identification, and genetic distance analyses expands the Neotropical representation of *Lispiviridae* and contributes to clarifying evolutionary relationships within *Mononegavirales*, reinforcing the relevance of metagenomic surveillance in periurban environments.

## 2. Materials and Methods

### 2.1. Sample Collection

Mosquitoes (*Anopheles* and *Culex*) were collected at the São Paulo Zoological Park Foundation (FPZSP), Brazil, currently designated as the DBB, located in the city of São Paulo, during different periods of 2020. The first sampling campaign occurred between May 5 and 7, when temperatures ranged from 11.1°C to 27.2°C, with fog on the 5th and 6th and light to moderate precipitation on the 7th, conditions typical of the transition from autumn to winter.

A second sampling effort was conducted between October 6 and 9, coinciding with the onset of the regional rainy season. During this period, environmental conditions were marked by increasing temperatures and relative humidity (average 81.3%), with daily thermal variation between 16.2°C and 37.4°C. Complementary meteorological observations from November and December indicated typical spring patterns, including heavy rainfall and thunderstorms, as reported by the Instituto Nacional de Meteorologia (INMET) [8].

Mosquito specimens were captured using 16 light‐bait traps (LBL) baited with carbon dioxide, distributed across eight distinct locations within the park. Collections took place under conditions of high temperature, elevated humidity, and intense rainfall factors known to favor mosquito abundance.

**FIGURE 1:**
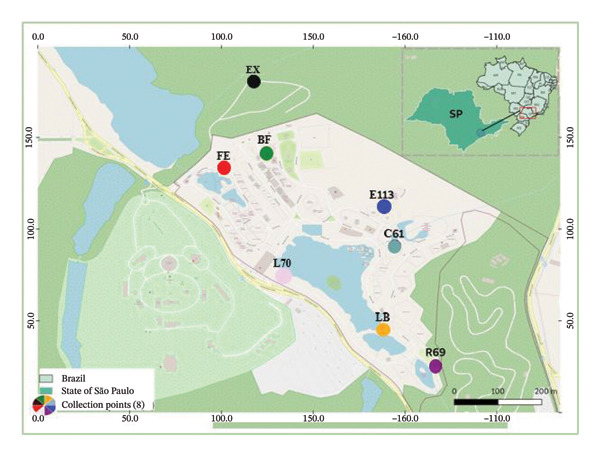
Geographic location of mosquito collection sites at the Diretoria de Biodiversidade e Biotecnologia (DBB), São Paulo, Brazil. The main map shows the study area with eight sampling points indicated by colored circles: Bird Forest (BF, green), Enclosure 113 (E113, blue), Corridor 61 (C61, gray), Lake Bridge (LB, orange), Enclosure 69 (R69, purple), Lake 70 (L70, pink), Flamingo Enclosure (FE, red), and Extra (EX, black). The inset map indicates the location of São Paulo state within Brazil. The scale bar represents 200 m.

The selection of collection points followed the methodology previously established by Guimarães et al. [6, 9]. At each site, two traps were installed at different vertical strata: one at ground level and another in the canopy. Distinct light sources were used as attractants, with white light applied to ground‐level traps and ultraviolet light to those positioned in the canopy. Figure 1 illustrates the spatial distribution of the eight sampling sites within the park, while Supporting Table S1 provides the geographic coordinates of each location and the vertical stratification of traps positioned at ground level (1.5 m) and in the canopy (6–10 m).

Traps operated for a 12 h interval, covering the period from dusk to dawn. Captured specimens were immediately transferred to labeled cryotubes, cryopreserved in liquid nitrogen while still alive, and transported to the laboratory, where they were stored at −80°C. Metadata associated with each sampling event, including climatic and environmental conditions, geographic location, sampling dates, number of collected tubes, and sampling effort, were systematically recorded.

The sampling design, which incorporated both the spatial distribution of sites and the placement of traps at different vertical strata, ensured comprehensive coverage of the study area. Standardized procedures for collection, preservation, and data documentation were consistently applied throughout the study.

### 2.2. Identification of Culicidae Species

The nonengorged adult females were identified based on morphological characteristics, using a stereomicroscope on a table refrigerated at −20°C. Taxonomic identification was carried out according to the identification keys described by Forattini [10], Consoli and Lourenço‐de‐Oliveira [11], and Lane [12], at the Vector Section of Pasteur Institute, São Paulo.

### 2.3. Preparation of Pools for Viral Metagenomic Analysis

Culicidae specimens were organized into pools containing 21 to 50 individuals, according to species, collection date, and sampling site, ensuring a minimum of 20 individuals per pool. Each pool was homogenized in Hanks’ balanced salt solution (HBSS) with 1.4 mm ceramic beads (Qiagen), using 100 μL of HBSS and 0.1 g of beads per specimen. The homogenization process was carried out using a FastPrep‐96 instrument (MP Biomedicals) at 1600 rpm for 90 s. Following homogenization, 500 μL of the resulting suspension was passed through 0.45 μm pore‐size membranes to eliminate cellular debris and other particulate material prior to subsequent analyses.

### 2.4. Nucleic Acid (DNA/RNA) Extraction

The filtered homogenate obtained as described above was subjected to enzymatic treatment to remove free nucleic acids, using a combination of 7 μL of TURBO DNase and 3 μL of RNase Cocktail Enzyme Mix (Thermo Fisher Scientific, USA). Total RNA was subsequently extracted using the Maxwell 16 Viral Total Nucleic Acid Purification Kit (Promega, USA), following the manufacturer’s instructions. Reverse transcription was performed using SuperScript IV (Thermo Fisher Scientific, USA) to synthesize first‐strand cDNA, followed by second‐strand synthesis using DNA Polymerase I Large (Klenow) Fragment (Promega, USA).

### 2.5. Preparation of Libraries for the Illumina Platform

The construction of the genomic library was performed using the Nextera XT DNA Library Preparation Kit (Illumina Inc., USA), following the manufacturer’s specifications. Sequencing was performed on the MiSeq sequencing platform (Illumina Inc., USA) at the Central Laboratory of the Hospital das Clínicas of the University of São Paulo.

### 2.6. Bioinformatics Analysis

The FastQC files were first subjected to a quality assessment using the FastQC tool [13] to verify the integrity and quality of the reads. Subsequently, low‐quality sequences were removed using the Trimmomatic algorithm [14]. The read set underwent de novo assembly using the rnaviralSPAdes assembler [15] with its default settings. The generated contigs were processed with CD‐HIT [16] to eliminate redundant sequences with ≥ 98% identity. Additionally, contigs shorter than 600 base pairs were discarded.

The taxonomic classification of contigs was initially performed using the DIAMOND algorithm [17] in BLASTx [18] mode against the NCBI RefSeq viral protein database and the Serratus [19] RdRp database. An E‐value threshold of 1E‐5 was applied to ensure high sensitivity while minimizing the occurrence of false positives. The binning performed with DIAMOND [17] was subsequently exported to the MEGAN [20] software for taxonomic assignment using the lowest common ancestor (LCA) algorithm, allowing hierarchical exploration of viral classifications. After this taxonomic curation step, complete or near‐complete genomes were aligned using the MUSCLE software, ensuring robust alignments for phylogenetic inferences [21].

### 2.7. Genome Characterization

Open reading frames (ORFs) were predicted using the ORFfinder v0.4.3 (accessed on May 02, 2025) [22] software, and the translated proteins were compared against reference viral protein databases through BLASTp (accessed on May 02, 2025) [23] searches, allowing the identification of protein similarities and the functional annotation of viral proteins.

For sequences corresponding to the RdRp, additional analyses were conducted to verify the presence of conserved catalytic signatures characteristic of viral polymerases. Conserved motifs and functional domains were identified using the tools Motif Finder (accessed on May 02, 2025) [24] and Conserved Domain Database (CDD) (accessed on May 03, 2025) [25]. In addition, the conserved catalytic motifs A, B, and C present in the palm domain of RdRp proteins were also characterized using PalmAnnot (accessed on May 04, 2025) [26]. This approach allowed the identification of typical RdRp catalytic signatures, which were subsequently validated as viral polymerize using the LucaProt server (accessed on May 04, 2025) [27]. Finally, multiple sequence alignment of motifs A, B, and C with homologous viral sequences from the families *Rhabdoviridae* and *Lispiviridae* was performed using the MUSCLE algorithm implemented in the Unipro UGENE platform [28] to confirm their conservation.

Despite these comprehensive in silico analyses, none of the viral sequences were validated by targeted RT‐PCR or Sanger sequencing. This study was based exclusively on metagenomic sequencing and de novo assembly. Genome completeness and viral identification were inferred from assembly metrics, functional annotation, identification of conserved motifs, genetic distance analyses, and phylogenetic inference, consistent with viruses of the order *Mononegavirales*, according to the International Committee on Taxonomy of Viruses (https://ictv.global/) [29].

Thus, the genomes described in this study represent assemblies derived from metagenomic data, supported by computational validation, without independent experimental confirmation.

### 2.8. Genetic Distance

The genetic distance between the sequences was estimated using the composite maximum likelihood model, incorporating rate heterogeneity via a gamma distribution and assessing the robustness of the estimates with a bootstrap of 1000 replicates. Similarity analysis was performed using the SDT software [30], employing pairwise comparisons between the sequences. The multiple alignments required for these comparisons were generated using the MUSCLE algorithm [21]. Based on the identity values obtained, phylogenetic relationships were inferred in the PHYLIP package [31] using the NEIGHBOR [32] module, employing the neighbor‐joining method. The resulting tree organized the sequences according to their evolutionary proximity, and the pairwise identity values were presented as frequency distributions in a graphical interface.

### 2.9. Phylogenetic Analysis

The phylogenetic tree was inferred using the maximum likelihood approach, using the most appropriate evolutionary model (VT + F + R10), previously estimated by the IQ‐TREE software. The statistical support of the branches was determined by a bootstrap test with 1000 repetitions [33]. The trees were visualized and edited using FigTree v1.4.2 software (https://tree.bio.ed.ac.uk/software/figtree/) [34].

All viral genome sequences generated in this study were deposited in GenBank under the following accession numbers: *AnRhabV-SP_01* (PX833262), *AnRhabV-SP_02* (PX833263), *Culex-SP_04* (PX833264), *CxRhabV-SP_05* (PX833265), *CxRhabV-SP_06* (PX833266), *CxRhabV-SP_08* (PX833267), *CxRhabV-SP_10* (PX833260), *CxRhabV-SP_11* (PX833261), *CuRhabV-SP_16* (PX833268), *CxLispV-SP_03* (PX833269), *CxLispV-SP_09* (PX833270), *CxLispV-SP_12* (PX833271), *CxLispV-SP_13* (PX833272), *CxLispV-SP_14* (PX833273), and *CxLispV-SP_15* (PX833274).

Raw sequencing reads were deposited under BioProject accession PRJNA1242354 and are associated with the following BioSample accession numbers: Mosq_lib21 (SAMN55864755), Mosq_lib22 (SAMN55864756), Mosq_lib23 (SAMN55864757), Mosq_lib28 (SAMN55864758), Mosq_lib41 (SAMN55864759), Mosq_lib43 (SAMN55864760), Mosq_lib46 (SAMN55864761), and Mosq_lib47 (SAMN55864762) (Supporting Table S2).

## 3. Results and Discussion

In this study, eight mosquito pools were analyzed (META 21, 22, 23, 28, 41, 43, 46, and 47) (Supporting Table S1), leading to the identification of 15 complete and partial viral genomes belonging to the families *Rhabdoviridae* and *Lispiviridae*. In addition to the taxonomic characterization of the detected viruses, understanding their ecological significance requires an examination of the environmental interfaces where vector–host interactions occur.

### 3.1. Expanded Ecological Context, Transmission Interface, and Spillover Implications

The mosquitoes analyzed in this study were collected from ecologically heterogeneous environments managed by the DBB, including aquatic bird enclosures, forest canopy sectors, mammal exhibits, and transitional corridors connecting different habitats. These microenvironments combine dense vegetation, permanent water bodies, accumulated organic matter, and high vertebrate diversity, creating favorable conditions for mosquito proliferation and for the maintenance of complex viral transmission cycles.

The maintenance of phylogenetically diverse species in close proximity, a common feature in zoological institutions, alters the natural flow of interaction between hosts and vectors. By simultaneously providing organisms from distinct habitats and biomes, these settings enhance the heterogeneity of hosts available to hematophagous vectors. Such a configuration may stabilize viral transmission within multispecies communities and create conditions conducive to the occurrence of interspecies spillover events [35].

The predominance of *Cx. chidesteri* in traps installed at both ground and canopy levels suggests ecological plasticity and potential involvement in bird‐associated transmission cycles. Species of the genus *Culex* are widely recognized for their ornithophilic behavior and their role in maintaining enzootic arbovirus cycles. The detection of *Culex (Mel.)* sp., belonging to the subgenus *Melanoconion*, which is frequently associated with sylvatic arbovirus transmission in the Neotropical region, further supports the hypothesis of viral circulation in forested environments.

The mosquito *Anopheles (Nys.) strodei* was collected in different vegetation strata of the FPZSP, including Bosque das Aves, Enclosure 69, Ponte do Lago, Flamingos Enclosure, Enclosure 113, Extra, Corridor 61, and Lago 70. This pattern differs from that observed for the other species collected, which showed a more restricted spatial distribution. These results suggest that *Anopheles (Nys.) strodei* has a wide distribution within the park and that these mosquitoes can adapt to different types of environments.

The observed vertical stratification is also ecologically relevant. The frequent capture of *Culex* species in canopy traps suggests interaction with arboreal birds, whereas collections at ground level may reflect transmission dynamics involving mammals. This vertical pattern may indicate the coexistence of distinct enzootic cycles within the same geographic area.

Anthropogenic environments, such as zoos, serve as convergence points for multiple potential hosts and vectors, fostering complex ecological interactions capable of sustaining viral dynamics that remain poorly characterized. Although this study did not identify direct evidence of spillover events, the spatial proximity between captive fauna, free‐living wildlife, and vector arthropods highlights the need for integrated surveillance strategies. This scenario is consistent with the One Health approach, particularly in contexts where biological connectivity increases opportunities for viral circulation [6].

Similar patterns of viral circulation and entomological surveillance in zoological settings have been reported at the Nashville Zoo at Grassmere, the Lisbon Zoo, and other institutions, where the combination of high host diversity, seminatural environments, and urban proximity favors the maintenance and detection of mosquito‐borne viruses [6, 36, 37]. These parallels reinforce the role of zoological parks as potential sentinel systems for emerging virus surveillance.

Collectively, the ecological diversity of the sampling sites, together with the species composition and vertical distribution of mosquitoes, supports the hypothesis that the detected virome reflects active and ecologically structured viral circulation. In environments characterized by high host density, multiple ecological interfaces, and pronounced environmental heterogeneity, viral maintenance may occur dynamically, with potential for occasional interspecific transmission events. These findings underscore the need for continuous virological and ecological surveillance in institutions that concentrate wildlife within urban contexts.

### 3.2. Viral Diversity

To better characterize the genomes of the 15 viral sequences in this study, they were compared with sequences deposited in the BLAST database (nt/aa). By means of RdRp similarity, phylogenetic analysis was performed, allowing for taxonomic classification and the naming of genomes, such as *Anopheles rhabdovirus* SP (*AnRhabV-SP*), *Culex rhabdovirus* SP (*CxRhabV-SP*), and *Culex lispivirus* (*CxLispV-SP*). Supporting Table S3 presents the comparative analysis of the viral genomes detected in Culicidae mosquito samples. Sequences *CxLispV-SP_03, _09, _14*, and *_15* were characterized by high coverage (> 94%) and identity above 87%, indicating high conservation relative to GenBank sequences. Samples *CxLispV-SP_12* and _13, despite sharing the same identity (87%), showed partial genomic coverage of 45% and 66%, respectively. In contrast, sequences such as *AnRhabV-SP_01* and *Culex-SP_04* exhibited low coverage (< 27%) and moderate identities (∼50%), suggesting the presence of more divergent viral variants.

Establishing these distinct levels of genomic conservation and coverage is essential for understanding the evolutionary dynamics of the identified viruses, providing valuable insights for phylogenetic studies and epidemiological surveillance.

Based on genome annotation, we characterized *AnRhabV-SP* and *CxRhabV-SP* (family *Rhabdoviridae*), as well as *CxLispV-SP* (family *Lispiviridae*). These viruses typically present from one (3′–L–5) to five ORFs arranged in the order 3′–N–X‐X–G–L–5′, which encode structural and nonstructural proteins essential for the viral replicative cycle. These ORFs include the nucleoprotein (N), hypothetical protein (X), glycoprotein (G), and large protein (L), the latter of which contains the functional domains of the RdRp (Supporting Table S4). The RdRp is responsible for transcription and replication of the viral genome. All characterized viruses possess single‐stranded, negative‐sense RNA genomes (–ssRNA), organized in a 3′ ⟶ 5′ orientation. Genome sizes ranged from approximately 6.149 to 13.713 kb for members of the *Lispiviridae* and from 5.140 to 11.373 kb for those in the *Rhabdoviridae* (Supporting Table S3) [4, 38, 39].

In addition to protein‐coding regions, these genomes contain conserved noncoding sequences at both ends: a 3′ leader and a 5′ trailer, which are critical for transcription initiation and genome replication. The presence and length of these regions were annotated for each viral sequence (Supporting Figure S1). Therefore, based on these results, the specific analyses for the sequences identified as belonging to the *Lispiviridae* and *Rhabdoviridae* families are presented below.

### 3.3. Identification of *Lispiviridae*

**FIGURE 2:**
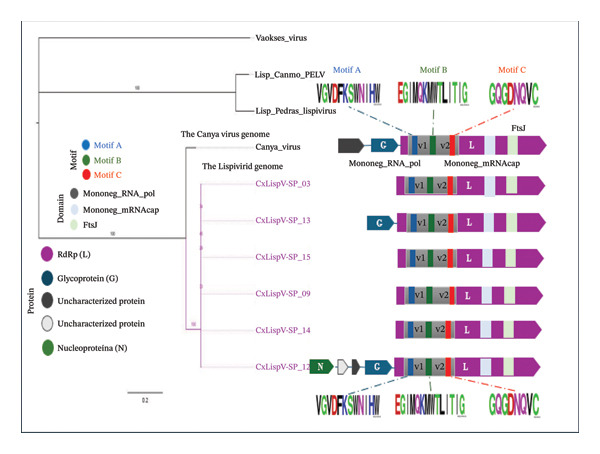
Representation of the *CxLispV-SP_03, 09, 12, 13, 14,* and *15* viral genomes in a phylogenetic tree with species from the *Lispiviridae* family. The clades of the tree in light plum color represent the *CxLispV-SP* sequences, while the branches in black indicate other species of the *Lispiviridae* family. The *CxLispV-SP* sequences group together in a highly sustained monophyletic clade (bootstrap ≥ 98) with Canya virus, within the *Lispiviridae* family, but are separate from the species previously described as *Lisp_Pedras_lispivirus* and *Lisp_Canmo_PELV*, suggesting a distinct viral lineage. The genomic organization of these sequences reveals the presence of conserved domains represented by dark gray (Mononeg_RNA_pol), light blue (Mononeg_mRNAcap), and light green (FtsJ). Conserved motifs are indicated by medium dark blue (motif A), dark lime green (motif B), and red (motif C). Structural proteins are color‐coded: nucleoprotein (N) in dark green, uncharacterized proteins in light and dark gray, glycoprotein (G) in dark teal, and RdRp (RdRp, L) in purple.

In the present study, we identified six viral sequences associated with mosquitoes of the genus *Culex*, ranging in size from 6149 to 13,713 nt. These sequences were designated *CxLispV-SP_03*, *CxLispV-SP_09, CxLispV-SP_12, CxLispV-SP_13, CxLispV-SP_14,* and *CxLispV-SP_15*. These genomes exhibited a negative‐sense (3′ to 5′) orientation, containing one to five ORFs (Figure 2) and were classified within the family *Lispiviridae* (Supporting Tables S3 and S4).

The *CxLispV-SP* sequences (*03, 09, 14,* and *15*) are characterized by the presence of a single ORF, which encodes the RdRp (L protein), with lengths ranging from 1880 to 2099 amino acids. This protein contains three conserved functional domains that are essential for the catalytic activity of RdRp.

The RdRp ORFs of sequences *CxLispV-SP_03* and _09, as well as *_13, _14*, and *_15*, exhibit amino acid sequence identities of 87.75%, 87.52%, and 87.57%, respectively, with the L protein (GenBank accession number (QRW41713.1) of the Canya virus, previously identified in metagenomic samples from *Culex tarsalis* mosquitoes in the United States in 2017 [40, 41].

Notably, the *CxLispV-SP_13* sequence contains an additional ORF, besides the L protein ORF, which encodes an uncharacterized protein and shows 52% coverage and 50.2% identity with a protein from the mosquito *Wyeomyia smithii* (GenBank accession number XP_055543613.1).

In contrast to the aforementioned viral genomes, the *CxLispV-SP_12* genome contains five distinct ORFs. These ORFs encode several proteins, including the nucleoprotein (N), the glycoprotein (G), the large protein (L), and two hypothetical proteins with currently unknown functions.

ORF1 shares 42% identity with a hypothetical protein (RP20_CCG024258) from *Aedes albopictus* (KXJ70270.1), isolated in China in 2015 [42]. ORFs 2 and 3 also encode uncharacterized proteins of unknown function.

### 3.4. Identification of *Rhabdoviridae*

Out of a total of 15 viral genomes identified in mosquitoes collected from FPZSP, eight were assigned to the family *Rhabdoviridae*. Among these, five (*AnRhabV-SP_01, AnRhabV-SP_02, CxRhabV-SP_05, CxRhabV-SP_06*, and *CxRhabV-SP_16*) correspond to partial genomes (5140–8263 bp), while three (*CxRhabV-SP_08, CxRhabV-SP_10*, and *CxRhabV-SP_11*) are complete (10,132–11,373 bp). These genomes contain one to five ORFs arranged in the order 3′‐N‐X‐X‐G‐L‐5′, with the exception of *CxRhabV-SP_11*, which exhibits the organization 3′‐N‐X‐M‐G‐L‐5′ (Supporting Tables S3 and S4).

**FIGURE 3:**
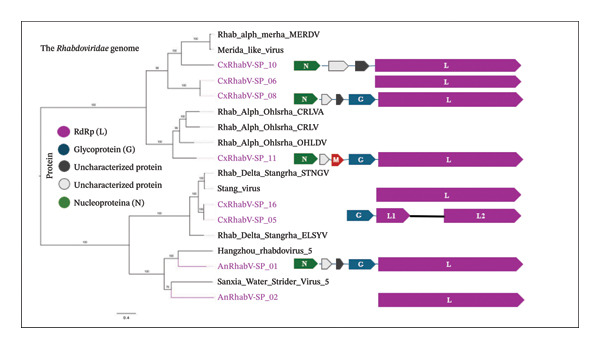
Phylogenetic tree with the viral genomes CxRhabV‐SP, AnRhabV‐SP, and the species of the *Rhabdoviridae* family. The light plum color represents CxRhabV‐SP and CxRhabV‐SP and black represents the species of the *Rhabdoviridae* family. *CxRhabV-SP_06*, _08, and _10 are grouped together with Merida_like_virus and MERDV; *CxRhabV-SP_11* forms a sister branch with the Rhab_Alph_Ohlsrha sequences (CRLVLA, CRLV, and OHLDV); *CxRhabV-SP_05* and _16 appear close to *Stangrha_STNGV* and *Stang_virus*, but form a distinct branch; *AnRhabV-SP_02* is positioned as a sister branch of Sanxia Water Strider Virus 5 and *AnRhabV-SP_01* appears close to Hangzhou rhabdovirus 5. The clades show strong bootstrap support (= 100 or ≤ 99 or ≤ 76). The proteins in the genome are color‐coded as follows: nucleoprotein (N) in dark green, matrix protein (M) in orange, uncharacterized proteins in light gray and dark gray, glycoprotein (G) in dark teal, and RNA‐dependent RNA polymerase (RdRp, L) in purple.

The ORFs encode structural and functional proteins, such as the nucleoprotein (N), matrix protein (M), glycoprotein (G), and large protein (L), in addition to hypothetical proteins (HP), whose function has not yet been characterized. The L protein contains conserved domains characteristic of RNA‐dependent RNA polymerases (RdRps) of the order *Mononegavirales*, specifically within the Mononeg_RNA_pol domain, where the A, B, and C catalytic motifs are located (Figure 3).

The RdRp sequences encoded by ORF5 of the *CuxLiV-06-SP*, *CuxLiV-08-SP,* and *CuxLiV-10-SP* isolates exhibit nucleotide sequence similarity levels of 99%, 99%, and 98%, respectively. These sequences share amino acid identities of 47.98%, 46.07%, and 49.86%, respectively, with the RdRp‐L protein of the Merida‐like virus (GenBank accession number: WPK42782.1), isolated from *Culex pipiens* in Georgia in 2018 [43]. Comparative BLASTx analysis confirmed that these ORFs encode the L protein of the *Riboviria* realm, reflecting the genomic diversity within this viral clade [40].

The viral genomes of *CxRhabV-SP_08* and *CxRhabV-SP_10* contained additional ORFs. ORF1, encoding the nucleoprotein (N), showed coverage levels of 96% and 51%, with sequence identities of 34.93% and 56.20%, respectively, when compared to nucleoproteins from *Stegomyia aegypti* (yellow fever mosquito, EAT48846.1) and *Wyeomyia smithii* (pitcher‐plant mosquito, XP_055543613.1) [44]. ORFs 2 and 3, present in both viral genomes, encode proteins of unknown function. ORF4, present only in *CxRhabV-SP_08* and responsible for encoding the glycoprotein (G), displayed 98% coverage and 44.36% identity to the corresponding protein of the Merida virus (GenBank accession number: UUG74169.1), isolated from *Culex pipiens* in China in 2018.

The genome of *CxRhabV-SP_11* showed 56.55% identity with the Ohlsdorf virus (GenBank accession number: UYE93948.1). Its genomic organization comprises multiple ORFs, consistent with the typical *Rhabdoviridae* structure, including the core genes N, P, M, G, and L. In addition to *CxRhabV-SP_11*, two additional partial genomes, *CxRhabV-SP_05* and *CxRhabV-SP_16*, were identified. These sequences exhibited high similarity, with 82.13% and 81.34% identity, respectively, to the Stang virus (GenBank accession numbers: QRW41829.1 and QRW41834.1).

Both sequences encode the L protein and demonstrated 99% coverage with 80.99% and 81.25% identity, respectively, in comparison with the L protein of the Stang virus (detected in *Culex erythrothorax*, QRW41834.1), which was first identified in the United States in 2017 [40]. Notably, *CxRhabV-SP_05* contains an additional ORF encoding a putative glycoprotein (G), exhibiting 100% coverage and 53.65% identity to the glycoprotein of Stang virus (QRW41833.1). In addition, CxRhabV‐SP_05 contains an additional RdRp, with a 394 nt region located between the ORFs encoding proteins of 442 aa and 1711 aa.

Further related sequences include *AnRhabV-SP_01* and *AnRhabV-SP_02*. The *AnRhabV-SP_01* genome shares the same ORF organization as *CxRhabV-SP_11*, consisting of five ORFs in the 3′‐N‐X‐M‐G‐L‐5′ arrangement. ORF1, which encodes a nucleoprotein (N), shares 100%% coverage and 35.48% identity with a homolog in *Culex rhabdo*‐like virus (*Culex quinquefasciatus*, YP_009388612.1), sampled in Australia: South Guildford in 2015. ORF2 encodes an uncharacterized protein of unknown function, whereas ORF3 showed similarity to the matrix protein (M) of Ohlsdorf virus (YP_010086748.1), with 84% coverage and 36.55% identity. ORF4, which encodes the glycoprotein G, displays 97% coverage and 30.73% identity with the Sanxia Water Strider Virus 5 glycoprotein (YP_009289351.1), isolated from an unidentified Gerridae host in China [45]. The L protein encoded by ORF5 shows 99% coverage and 50% identity with the corresponding protein of Hangzhou rhabdovirus 5 (GenBank accession number: UHK03252.1), isolated from *Hydrellia griseola* in China in 2013 [45]**.**

In contrast, the *AnRhabV-SP_02* genome contains a single ORF, which encodes the large protein (L). This ORF shows 96% coverage and 41.64% identity with the L protein of Sanxia Water Strider Virus 5 (GenBank accession number: YP_009289352.1), also isolated from *Hydrellia griseola* in China in 2013 [45].

### 3.5. Analysis of Domain and Conserved Motifs

To more effectively explore the differences among the *CxLispV-SP, AnRhabV-SP*, and *CxRhabV-SP* sequences, we analyzed the RdRp protein from viral sequences belonging to the families *Lispiviridae* (*CxLispV-SP_03*, *CxLispV-SP_09*, *CxLispV-SP_12*, *CxLispV-SP_13*, *CxLispV-SP_14*, and *CxLispV-SP_15*) and *Rhabdoviridae* (*AnRhabV-SP_01*, *AnRhabV-SP_02*, *CxRhabV-SP_05*, *CxRhabV-SP_06*, *CxRhabV-SP_08*, CxRhabV‐SP_09, *CxRhabV-SP_10*, *CxRhabV-SP_11*, and *CxRhabV-SP_16*). This protein, expressed as a single large polypeptide encoded by the L gene, constitutes the main catalytic subunit of the RNA polymerase in viruses of the order *Mononegavirales* and contains a set of functional domains specialized in the post‐transcriptional modification of viral RNA [4, 38, 39].

The identified domains include *Mononegavirales* RdRp (Mononeg_RNA_pol), followed by its respective structural motifs; *Mononegavirales* mRNA‐capping region V (Mononeg_mRNAcap); FtsJ‐like methyltransferase (FtsJ); virus‐capping methyltransferase, connector domain (Methyltrans_Mon_1st); virus‐capping methyltransferase, MT domain (Methyltrans_Mon_2nd); virus‐capping methyltransferase, C‐terminal (Methyltrans_Mon_3rd); GDSL‐like lipase/acylhydrolase (Lipase_GDSL); and Gal4‐like dimerization domain (Gal4_dimer). A detailed description of these domains, including Pfam identifiers, genomic positions, and i‐Evalue, is provided in Supporting Table S5.

The functions of the L protein (RdRp) domains highlight the complexity of the replicative machinery of viruses belonging to the order *Mononegavirales*. The Mononeg_RNA_pol domain (PF00946) is directly involved in viral RNA synthesis, whereas Mononeg_mRNAcap (PF14318) participates in processes associated with 5′ cap formation, which is essential for RNA stability and translation [4, 38, 39].

The FtsJ methyltransferase (PF01728) exhibits structural homology to S‐adenosyl‐L‐methionine (SAM)‐dependent enzymes associated with RNA methylation reactions, including modifications at the 5′ end [49–51]. The Methyltrans_Mon_1st (PF21080), Methyltrans_Mon_2nd (PF14314), and Methyltrans_Mon_3rd (PF21081) correspond to modules related to viral methyltransferase activity, associated with viral messenger RNA processing and maturation steps, including methylation events that contribute to cap formation and stability [46–48].

The Lipase_GDSL domain (PF00657), identified in one of the sequences, is associated with lipolytic and esterase activities and may be related to processes involving interaction with cellular membranes [52–54]. The Gal4_dimer domain (PF03902), associated with protein dimerization, suggests potential involvement in protein–protein interactions [55]. Finally, the Lipoprotein_15 domain (PF03640) indicates possible interaction with host lipid components.

Collectively, these domains reflect functions related to viral RNA replication, modification, and stability, as well as potential structural interactions with cellular components.

**FIGURE 4:**
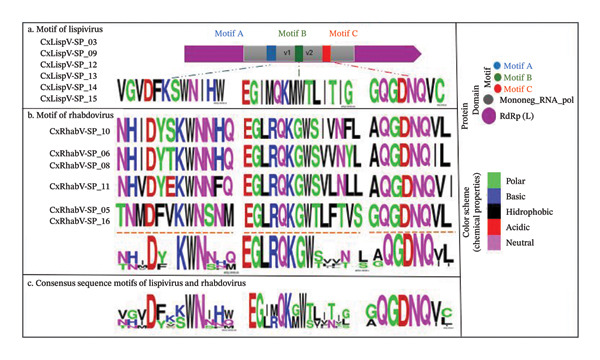
Conserved motifs of RdRp (L) in CxLispV‐SP and CxRhabV‐SP: comparison between *Lispiviridae* and *Rhabdoviridae* families. (a) Alignment of motifs A, B, and C of the coding region of the RdRp protein (*Mononeg_RNA_pol*) of viruses *CxLispV-SP_03*, _09, _12, _13, _14, and _15, grouped in the *Lispiviridae* family. (b) Corresponding alignment of the same motifs in the *CxRhabV-SP_05*, _06, _08, _10, _11, and _16 sequences, belonging to *the Rhabdoviridae* family, and the consensus between them. (c) Consensus sequence generated by comparing the motifs of the two families. The domains were organized based on the annotation of the L protein, including the functional regions corresponding to the catalytic motifs A (blue), B (green), and C (red), which are highly conserved in viruses of the order *Mononegavirales*. The colors of the amino acids reflect their chemical properties: green (polar), blue (basic), gray (hydrophobic), red (acidic), and black (neutral).

**FIGURE 5:**
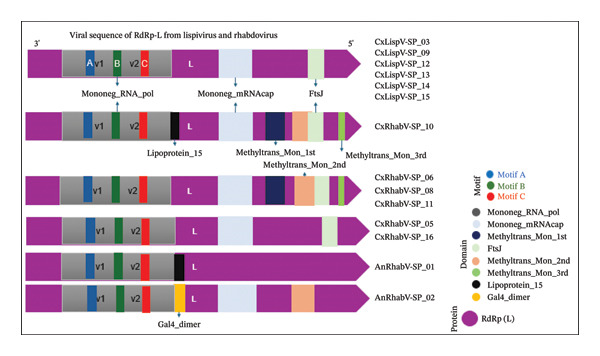
RdRp (L) protein domains in *CxLispV-SP, AnRhabV-SP,* and *CxRhabV-SP*: comparison between *Lispiviridae* and *Rhabdoviridae* families. Schematic representation of the conserved domains of the L protein (RdRp) in purple identified in the viruses *CxLispV-SP* (*Lispiviridae*) and *CxRhabV-SP* and *AnRhabV-SP* (*Rhabdoviridae*). The genomic orientation is represented from 3′ to 5′. The CxLispV‐SP viruses present the typical domains of the *Mononegavirales* order, such as dark gray (Mononeg_RNA_pol), light blue (Mononeg_mRNAcap), and light green (FtsJ), as well as the medium dark blue (motif A), dark lime green (motif B), and red (motif C) catalytic motifs, conserved in the viral polymerase, with the exception of AnRhabV‐SP. In contrast, members of the *Rhabdoviridae* family share these domains, but exhibit greater diversity, with the presence of additional regions such as dark blue (Methyltrans_Mon_1st), light orange (Methyltrans_Mon_2nd), green (Methyltrans_Mon_3rd), black (Lipoprotein_15), and gold (Gal4_dimer). This structural variation may reflect functional and phylogenetic differentiations between the viral families analyzed.

The functional domain Mononeg_RNA_pol (PF00946) was identified in all fourteen viral sequences analyzed. Within this domain, three conserved motif regions were detected, comprising 12, 14, and 8 amino acids, corresponding to motifs A, B, and C, with the following consensus sequences: xxxDxxxWNxxx, EGxxQKxW (*n* = 6), and xQGDNQxx, respectively. The only exceptions were sequences *AnRhabV-SP_01* and *AnRhabV-SP_02*, in which these motifs were not detected. The motifs and domains are presented in Figures 4 and 5.

The Mononeg_mRNAcap (PF14318) domain was present in most sequences, indicating maintenance of the viral mRNA capping machinery. Differences in the presence of the FtsJ domain and additional methyltransferase modules (Methyltrans_Mon_1st, Methyltrans_Mon_2nd, and Methyltrans_Mon_3rd) revealed structural variation between *Lispiviridae* and *Rhabdoviridae*, particularly among longer sequences (Supporting Table S5).

Functional analysis of the conserved motifs reinforced the integrity of the catalytic site. Motif A (xxxDxxxWNxxx) is associated with interaction with the RNA template strand, with residue D (Asp) playing a role in substrate positioning and residue tryptophan (W) contributing to stabilization of the enzyme–RNA interaction [56–58]. Motif B (EGxxQKxW (*n* = 6)) contributes to structural organization of the catalytic site and alignment of the template strand, with functional involvement of residues Q (glutamine), K (lysine), and W [58]. Motif C (xQGDNQxx), located in the palm domain, constitutes a central catalytic element of the polymerase and is involved in coordination of essential metal ions (Mg^2+^ or Mn^2+^), with residue D being critical for enzymatic activity [58]**.**

The combined presence of motifs A, B, and C is characteristic of the L protein of viruses within the order *Mononegavirales*, reflecting high structural and functional conservation [57–60]. In CxLispV‐SP sequences, motifs A (VGVDFKSWNIHW), B (EGIMQKMWTLITIG), and C (GQGDNQVC) were identical to those described for Canya virus. In contrast, *CxRhabV-SP* sequences exhibited minor variations in these motifs, differing by one to three residues depending on the phylogenetic grouping analyzed (Supporting Figure S2 and Table S6), without evidence of disruption of the catalytic core.

Overall, the domain composition and conserved catalytic motifs observed in the analyzed sequences are consistent with their classification within the order *Mononegavirales* and support the structural conservation of the L protein, while highlighting differences in methyltransferase‐associated regions between *Lispiviridae* and *Rhabdoviridae*.

### 3.6. Genetic Divergence Patterns

Based on the SDT v1.2 identity matrix, which calculates pairwise nucleotide identities among viral species [30], the RdRp sequences revealed distinct identity patterns within *Lispiviridae* and *Rhabdoviridae* (Supporting Figure S3). Within *Lispiviridae*, *CxLispV-SP* sequences (*CxLispV-SP_03*, *_09*, *_12*, *_13*, *_14*, and *_15*) shared high identity with Canya virus, exhibiting high intragroup identity (> 88%), consistent with closely related variants and supporting their classification within a cohesive viral complex, potentially representing a distinct group within the genus *Lispivirus*. In contrast, Vaokses virus, *Lisp_Canmo_PELV*, and *Lisp_Pedras_lispivirus* formed a separate lineage (< 63% identity relative to the *CxLispV-SP* group), reinforcing their genetic distinctiveness.

In contrast, Vaokses virus, *Lisp_Canmo_PELV*, and *Lisp_Pedras_lispivirus* formed a separate lineage (< 63% identity relative to the *CxLispV-SP* cluster), reinforcing their genetic distinctiveness.

In *Rhabdoviridae*, pairwise identities ranged from 26% to 100%, revealing substantially higher genetic dispersion. Four identity‐based groups were observed: (i) a highly conserved clade (> 85%) including *Rhabd_Delta-*related viruses together with *CxRhabV-SP_05* and *CxRhabV-SP_16*; (ii) an intermediate group (70%–85%) comprising *AnRhabV-SP_01*, *AnRhabV-SP_02*, and Sanxia Water Strider Virus 5; (iii) a moderately related Alpharhabdovirus‐associated cluster (55%–70%); and (iv) a highly divergent group (< 55%, with some values < 30%), including *CxRhabV-SP_06*, *CxRhabV-SP_08*, *CxRhabV-SP_10*, and Merida‐like viruses.

The broader identities spectrum observed in *Rhabdoviridae* compared to the more conserved pattern in *Lispiviridae* suggests a broader evolutionary diversification pattern within rhabdoviruses, potentially influenced by wider host range and ecological adaptability. Collectively, these findings support the L protein as a reliable evolutionary marker for taxonomic and phylogenetic inference within the order *Mononegavirales*.

From a biogeographic perspective, insect‐associated rhabdoviruses have been increasingly reported across the American continent, including Merida‐like viruses described in Mexico [61] and putative rhabdoviral sequences identified in mosquito viromes from the Brazilian Pantanal [62]**.** These viruses typically exhibit canonical *Mononegavirales* genome organization and cluster according to vector association and ecological niche. The genetic divergence observed here is consistent with previously reported patterns of insect‐associated rhabdovirus diversity across distinct ecological regions of the Americas. Within this continental framework, the sequences identified in São Paulo expand the known diversity of insect‐associated *Mononegavirales* in the Neotropics and suggest the circulation of additional undescribed lineages.

### 3.7. Phylogenetic Inferences

**FIGURE 6:**
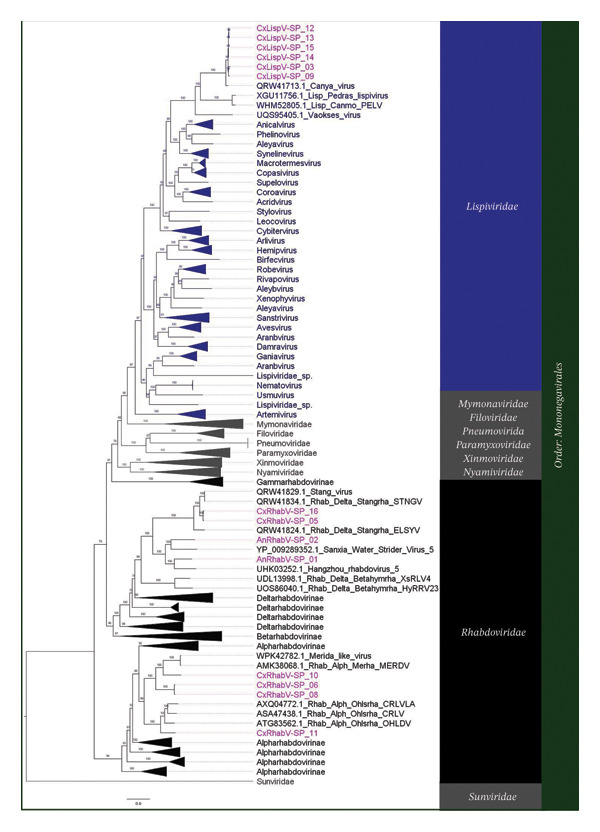
RdRp phylogenetic tree of the order *Mononegavirales*. The phylogeny was reconstructed based on 196 RdRp amino acid sequences using the maximum likelihood (ML) method. Branch support was assessed using bootstrap analysis, with values indicated at the nodes. The viral sequences identified in this study are highlighted in magenta. The family *Lispiviridae* is indicated in dark blue, whereas the family *Rhabdoviridae* is shown in black, including the subfamilies *Alpharhabdovirinae*, *Betarhabdovirinae*, *Gammarhabdovirinae*, and *Deltarhabdovirinae*. The remaining *Mononegavirales* families are shown in gray. Collapsed clades represent clusters of multiple sequences. The scale bar indicates the expected number of amino acid substitutions per site.

The phylogenetic analysis of the viral sequences *CxLispV-SP*, *AnRhabV-SP,* and *CxRhabV-SP* was based on a total of 196 RdRp coding sequences representing viruses from the order *Mononegavirales*, including the newly identified sequences. The inferred tree shows the distribution of the new sequences into six clades corresponding to established families within the order *Mononegavirales*, such as *Lispiviridae* and *Rhabdoviridae*. Statistical support for the groupings was assessed using bootstrap values (Figure 6).

The sequences *CxLispV-SP_03, _09, _12, _13, _14,* and *_15* clustered with a viral sequence closely related to Canya virus (QRW41713.1) and were clearly separated from other described species within the *Lispiviridae* family, such as *Lisp_Pedras_lispivirus* (XGU11756.1) and *Lisp_Canmo_PELV* (WHM52805.1). These sequences formed a well‐supported monophyletic clade with high internal *identity* and exhibited low similarity (< 70%) to other viruses within the genus *Canmovirus*, suggesting they may represent a putative novel viral species within the family.

Similarly, the *CxRhabV-SP_16* and *CxRhabV-SP_05* sequences grouped with *Rhab_Delta_Stangrha_STNGV* and Stang_virus (both represented by QRW41834.1), sharing 81.25% and 76.77% sequence identity, respectively. These viruses are members of the genus *Stangrhavirus* within the subfamily *Deltarhabdovirinae* and formed a strongly supported phylogenetic clade.

The *AnRhabV-SP_02* sequence clustered closely with Sanxia_Water_Strider_Virus_5 (YP_009289352.1), although it occupied a distinct phylogenetic branch supported by high bootstrap values, sharing 50% sequence identity. In contrast, *AnRhabV-SP_01* formed an isolated branch, positioned near Hangzhou_rhabdovirus_5 (UHK03252.1), with low sequence identity (30.73%), supporting its distinction as a highly divergent lineage and potentially representing a putative novel species.

Additionally, the *CxRhabV-SP_10*, *CxRhabV-SP_06,* and *CxRhabV-SP_08* sequences clustered near *Merida_like_virus* (WPK42782.1) and *Rhab_Alph_Merha_MERDV* (AMK38068.1), aligning them with members of the genus *Merhavirus* in the subfamily *Alpharhabdovirinae*. The *CxRhabV-SP_11* sequence showed phylogenetic affinity to *Rhab_Alph_Ohlsrha_CRLVLA/CRLV/OHLDV* (AXQ04772.1, ASA47438.1, and ATG83562.1), forming a distinct basal branch with 56.55% sequence identity, which may indicate the presence of a putative novel viral lineage within the genus *Ohlsrhavirus*.

Therefore, the results of the phylogenetic analysis indicate that the sequences identified in this study are distributed in well‐defined and strongly supported clades, compatible with the *Rhabdoviridae* family (subfamilies *Alpharhabdovirinae* and *Deltarhabdovirinae*; genera *Ohlsrhavirus*, *Merhavirus*, and *Stangrhavirus*) and with the *Lispiviridae* family (genus *Canmovirus*).

Among the *Lispiviridae*‐related sequences (*CxLispV-SP_03, _09, _12, _13, _14,* and *_15*), we identified a cluster phylogenetically close to Canya virus, but distant from known species such as *Lisp_Pedras_lispivirus* (XGU11756.1) and *Lisp_Canmo_PELV* (WHM52805.1), suggesting a putative novel species. However, no experimental data currently confirm their pathogenicity. *Lispiviridae* viruses infect a wide range of hosts, including insects of agricultural and health importance, such as mosquitoes. Some species are known to affect plant health via vectors such as the whitefly (*Bemisia tabaci*) [63], and others have been found in grasshoppers (*Acrida cinerea*) and bedbugs (*Erthesina fullo*) [64]. Additional hosts include the wasp *Anisopteromalus calandrae*, a parasite of rice weevils, impacting grain storage [65]. *Lispiviridae* members have also been detected in arachnids, birds, and various hematophagous arthropods such as ticks and mosquitoes [38], as well as termites [66], microcrustaceans [41], and organisms from the orders Odonata, Coleoptera, and even nematodes [67].

The remaining sequences, *CxRhabV-SP_16*, _*05, _06, _08, _10,* and *_11* and *AnRhabV-SP_01* and _02 were grouped within the *Rhabdoviridae* family, subfamilies *Alpharhabdovirinae* and *Deltarhabdovirinae*, and classified under the genera *Ohlsrhavirus*, *Merhavirus*, and *Stangrhavirus*. Notably, viruses of the genus *Ohlsrhavirus* have been identified in Culicidae mosquitoes worldwide, including in Hungary [68], Asia [69], Japan [70], Australia [71], California [72], and Brazil [73]. *Ohlsdorf virus* (*Ohlsrhavirus ohlsdorf*) was first described in *Ochlerotatus cantans* in Germany, with multiple detections between 2013 and 2015 [74].

## 4. Conclusions

This study provides robust insights into the diversity, genomic organization, and evolutionary relationships of viruses belonging to the families *Lispiviridae* and *Rhabdoviridae* identified in mosquitoes (Diptera: Culicidae, *Cx. chidesteri,* and *Cx. renatoi*). Through metagenomic sequencing, comparative genomic analyses, phylogenetic reconstruction, nucleotide identity assessment, and structural characterization of the RdRp protein, multiple insect‐associated *Mononegavirales* circulating in the Neotropics were identified and characterized. The combined evidence from monophyletic clustering, sequence divergence thresholds, conserved catalytic motifs, and domain architecture supports the taxonomic placement of the identified viruses and sustains the recognition of a putative novel species within *Lispiviridae*, closely related to Canya virus. Overall, this work identifies and characterizes the genomic diversity and evolutionary structuring of mosquito‐associated viruses, reinforcing the relevance of integrative metagenomic approaches for refining viral taxonomy and elucidating diversification patterns within negative‐sense RNA viruses.

## Author Contributions

Lilian de Oliveira Guimarães: conceptualization, data curation, formal analysis, methodology, writing–original draft, and writing–review and editing. Roseane da Silva Couto: formal analysis and writing–review and editing. Geovani de Oliveira Ribeiro: formal analysis and writing–review and editing. Vanessa dos Santos Morais: formal analysis and writing–review and editing. Juliana Telles‐de‐Deus: formal analysis and writing–review and editing. Vanessa Christe Helfstein: formal analysis and writing–review and editing. Jesus Maia dos Santos: formal analysis and writing–review and editing. Endrya do Socorro Foro Ramos: formal analysis and writing–review and editing. Ramendra Pati Pandey: writing–review and editing. Vera Lucia Fonseca de Camargo‐Neves: data curation, funding acquisition, resources, and writing–review and editing. Antonio Charlys da Costa: conceptualization, formal analysis, methodology, project administration, supervision, writing–original draft, and writing–review and editing. Karin Kirchgatter: conceptualization, funding acquisition, project administration, resources, supervision, writing–original draft, and writing–review and editing. Élcio Leal: project administration, supervision, writing–original draft, and writing–review and editing.

## Funding

The authors declare financial support was received for the research, authorship, and/or publication of this article. This research was benefited from the State Research Institutes Modernization Program, supported by Fundação de Amparo à Pesquisa do Estado de São Paulo (2017/50345‐5). Lilian de Oliveira Guimarães was supported by a postdoctoral fellowship (FAPESP 2018/16232‐1). Karin Kirchgatter is a CNPq research fellow (303040/2025‐4). Élcio Leal is supported by CNPq grant number: 305566/2025‐3.

## Conflicts of Interest

The authors declare no conflicts of interest.

## Supporting Information

Additional supporting information can be found online in the Supporting Information section.

## Supporting information


**Supporting Information 1** Figure S1: genomic organization of viruses from the *Lispiviridae* and *Rhabdoviridae* families identified in mosquitoes. Schematic representation of the genomic structure of viral species detected in mosquito samples, highlighting the main coding regions and their transcriptional orientation. Viruses classified as *Lispiviridae* (*CxLispV-SP_03*, _*09, _12, _13, _14,* and *_15*) are shown on the left, while viruses classified as *Rhabdoviridae* (*AnRhabV-SP_01* and _02; *Culex-SP_04*; CxRhabV‐SP_01, _02, _04, _05, _06, _08, _10, _11, and _16) are shown on the right. Coding sequences are represented by arrows in the 3′ ⟶ 5′ direction, corresponding to the negative‐sense RNA genome orientation. Colors indicate protein classes: dark green (N, nucleoprotein), orange (matrix protein, M), blue (G, glycoprotein), purple (L, RdRp–RdRp), and gray tones (uncharacterized proteins). Leader and trailer regions are indicated with their respective nucleotide lengths, illustrating variability among species. The nucleotide scale (nt) allows comparison of genome sizes across viruses. Differences in gene content—such as the presence or absence of N and G in some *Lispiviridae* or incomplete genomic ends in several *Rhabdoviridae*—reflect distinct evolutionary trajectories and may influence viral replication, host adaptation, and ecological interactions.

**Supporting Information 2** Figure S2: distribution and conservation of functional motifs in RdRp. Graphical representation of motifs A, B, and C using WebLogo, highlighting subtle variations in positions (A: 1–3, 5–7, 10–12; B: 3, 4, 7, 9–17; C: 2–6). The height of each letter represents the relative frequency of the amino acid at that position, while the total height of the stack indicates the degree of sequence conservation. Across the three motifs, highly conserved residues were observed, suggesting essential functional roles. In motif A, residues D, K, and W are prominent, directly linked to the catalytic activity of viral proteins. In motif B, the conservation of E, G, R, Q, and K implies an important role in nucleic acid binding and enzyme structural integrity. In motif C, the preserved residues Q, G, D, N, and Q indicate a potentially critical region associated with the active site of the viral RNA‐dependent RNA polymerase.

**Supporting Information 3** Figure S3: identity analysis of the RdRp (L) protein between viruses from the *Lispiviridae* and *Rhabdoviridae* families. Identity matrix generated with the sequence. Demarcation Tool (SDT) software, representing the CxLispV‐SP, CxRhabV‐SP, and CxRhabV‐SP sequences of the L protein (RdRp) of viruses from the (a) *Lispiviridae* and (b) *Rhabdoviridae* families. The color gradient reflects the levels of identity, ranging from blue (low identity) to red (high identity), according to the lateral scale. (a) In *Lispiviridae*, we observed two main groupings: group 1, with the viruses *CxLispV-SP_03, 09, 13, 15,* and *12* and Canya virus, showing high nucleotide identity (> 80%), indicating a close evolutionary relationship. Group 2, made up of viruses such as *Lisp_Pedras_lispivirus* and *Lisp_Canmo_PELV*, with identity lower than 60% in relation to group 1, suggests a distinct lineage. (b) In *Rhabdoviridae*, four distinct groups were delimited: group 1, made up of *CxRhabV-SP_05* and *16*, and Stang virus, with identity > 80%; group 2, with viruses such as *CxRhabV-SP_08*, *CxRhabV-SP_06*, *CxRhabV-SP_10,* and *Rhab_Delta_Stangha_ELSVV*, with identity between ∼70 and 75%; group 3, including *AnRhabV-SP_01*, Sanxia Water Strider Virus 5, Hangzhou rhabdovirus 5, and representatives of the genus *Alpharhabdovirus*, with moderate identity (50%–65%); group 4, represented by more divergent viruses such as Merida virus, *Rhab_Alph_Metra_MERV*, and *Rhab_Alph_Oshima_OHLVD*, with less than 50% identity, showing greater phylogenetic distance.

**Supporting Information 4** Table S1: composition of metagenomic pools based on taxonomic and geospatial criteria. Specimens were collected from ground and canopy strata across eight sites at the FPZSP.

**Supporting Information 5** Table S2: sequencing and assembly metrics of metagenomically assembled viral genomes identified in mosquito samples.

**Supporting Information 6** Table S3: similarity analysis of viral genomes identified in mosquito samples using BLASTp.

**Supporting Information 7** Table S4: comparative analysis of the genomes and ORF structures of CxLispV and AnRhabV.

**Supporting Information 8** Table S5: Pfam‐predicted functional domains, genomic positions, and statistical significance (i‐Evalue) identified in RdRp (L protein) sequences from mosquito‐associated viruses.

**Supporting Information 9** Table S6: distribution and conservation of functional motifs in RdRp.

## Data Availability

The viral sequences identified in this study have been deposited in the GenBank database and will be released upon publication of this manuscript. All other data related to the methodology and analysis are provided in the supporting files.
