# Supplementary material for: Putative Novel Viruses in the Families Lispiviridae and Rhabdoviridae Detected in Culex and Anopheles Mosquitoes Collected at the São Paulo Zoo
Source: Adv Virol. 2026 Jun 29;2026:8104754. doi: 10.1155/av/8104754 (PMC13315819; doi:10.1155/av/8104754)
Supplement: Supplementary file 1 — Supporting Information 1 Figure S1: genomic organization of viruses from the Lispiviridae and Rhabdoviridae families identified in mosquitoes. Schematic representation of the genomic structure of viral species detected in mosquito samples, highlighting the main coding regions and their transcriptional orientation. Viruses classified as Lispiviridae (CxLispV-SP_03, _09, _12, _13, _14, and _15) are shown on the left, while viruses classified as Rhabdoviridae (AnRhabV-SP_01 and _02; Culex-SP_04; CxRhabV‐SP_01, _02, _04, _05, _06, _08, _10, _11, and _16) are shown on the right. Coding sequences are represented by arrows in the 3′ ⟶ 5′ direction, corresponding to the negative‐sense RNA genome orientation. Colors indicate protein classes: dark green (N, nucleoprotein), orange (matrix protein, M), blue (G, glycoprotein), purple (L, RdRp–RdRp), and gray tones (uncharacterized proteins). Leader and trailer regions are indicated with their respective nucleotide lengths, illustrating variability among species. The nucleotide scale (nt) allows comparison of genome sizes across viruses. Differences in gene content—such as the presence or absence of N and G in some Lispiviridae or incomplete genomic ends in several Rhabdoviridae—reflect distinct evolutionary trajectories and may influence viral replication, host adaptation, and ecological interactions. [file AV-2026-8104754-s008.docx]

**Supplementary Materials**

**Figure S1
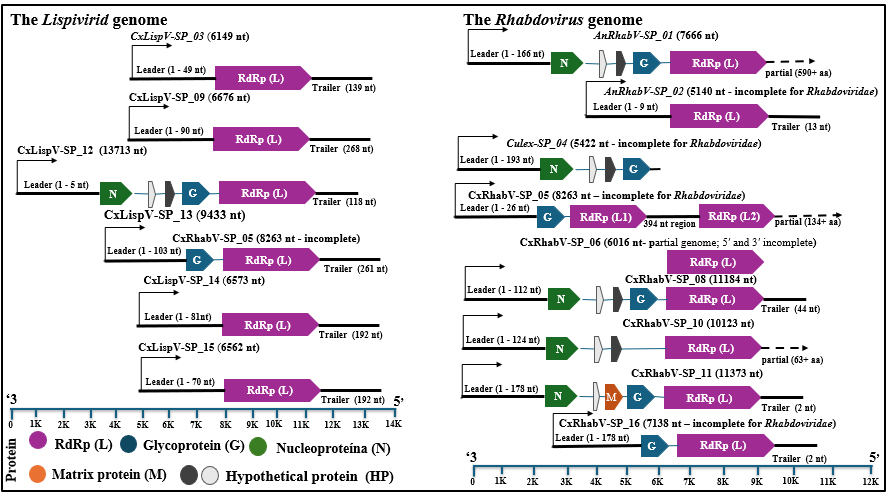
**

**Figure S1 - Genomic organization of viruses from the *Lispiviridae* and *Rhabdoviridae* families identified in mosquitoes.** Schematic representation of the genomic structure of viral species detected in mosquito samples, highlighting the main coding regions and their transcriptional orientation. Viruses classified as *Lispiviridae* (*CxLispV-SP_03*, _09, _12, _13, _14, and _15) are shown on the left, while viruses classified as *Rhabdoviridae* (*AnRhabV-SP_01* and _02; *Culex-SP_04*; CxRhabV‑SP_01, _02, _04, _05, _06, _08, _10, _11, and _16) are shown on the right. Coding sequences are represented by arrows in the 3′→5′ direction, corresponding to the negative‑sense RNA genome orientation. Colors indicate protein classes: dark green (N, nucleoprotein), orange (Matrix protein, M), blue (G, glycoprotein), purple (L, RdRp– RdRp), and gray tones (uncharacterized proteins). Leader and trailer regions are indicated with their respective nucleotide lengths, illustrating variability among species. The nucleotide scale (nt) allows comparison of genome sizes across viruses. Differences in gene content—such as the presence or absence of N and G in some *Lispiviridae*, or incomplete genomic ends in several *Rhabdoviridae*—reflect distinct evolutionary trajectories and may influence viral replication, host adaptation, and ecological interactions.
