# Supplementary material for: Putative Novel Viruses in the Families Lispiviridae and Rhabdoviridae Detected in Culex and Anopheles Mosquitoes Collected at the São Paulo Zoo
Source: Adv Virol. 2026 Jun 29;2026:8104754. doi: 10.1155/av/8104754 (PMC13315819; doi:10.1155/av/8104754)
Supplement: Supplementary file 2 — Supporting Information 2 Figure S2: distribution and conservation of functional motifs in RdRp. Graphical representation of motifs A, B, and C using WebLogo, highlighting subtle variations in positions (A: 1–3, 5–7, 10–12; B: 3, 4, 7, 9–17; C: 2–6). The height of each letter represents the relative frequency of the amino acid at that position, while the total height of the stack indicates the degree of sequence conservation. Across the three motifs, highly conserved residues were observed, suggesting essential functional roles. In motif A, residues D, K, and W are prominent, directly linked to the catalytic activity of viral proteins. In motif B, the conservation of E, G, R, Q, and K implies an important role in nucleic acid binding and enzyme structural integrity. In motif C, the preserved residues Q, G, D, N, and Q indicate a potentially critical region associated with the active site of the viral RNA‐dependent RNA polymerase. [file AV-2026-8104754-s001.docx]

**Figure S2**
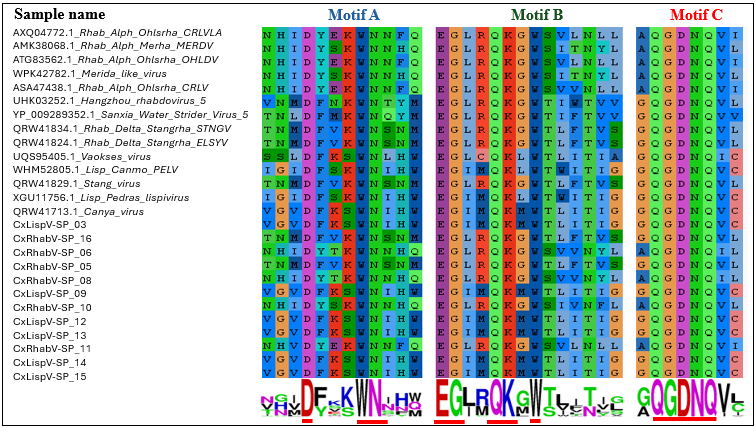


**Figure S2. Distribution and Conservation of Functional Motifs in RdRp.** Graphical representation of motifs A, B, and C using WebLogo, highlighting subtle variations in positions (A: 1–3, 5–7, 10–12; B: 3, 4, 7, 9–17; C: 2–6). The height of each letter represents the relative frequency of the amino acid at that position, while the total height of the stack indicates the degree of sequence conservation. Across the three motifs, highly conserved residues were observed, suggesting essential functional roles. In Motif A, residues D, K, and W are prominent, directly linked to the catalytic activity of viral proteins. In Motif B, the conservation of E, G, R, Q, and K implies an important role in nucleic acid binding and enzyme structural integrity. In Motif C, the preserved residues Q, G, D, N, and Q indicate a potentially critical region associated with the active site of the viral RNA-dependent RNA polymerase.
