# Supplementary material for: Putative Novel Viruses in the Families Lispiviridae and Rhabdoviridae Detected in Culex and Anopheles Mosquitoes Collected at the São Paulo Zoo
Source: Adv Virol. 2026 Jun 29;2026:8104754. doi: 10.1155/av/8104754 (PMC13315819; doi:10.1155/av/8104754)
Supplement: Supplementary file 3 — Supporting Information 3 Figure S3: identity analysis of the RdRp (L) protein between viruses from the Lispiviridae and Rhabdoviridae families. Identity matrix generated with the sequence. Demarcation Tool (SDT) software, representing the CxLispV‐SP, CxRhabV‐SP, and CxRhabV‐SP sequences of the L protein (RdRp) of viruses from the (a) Lispiviridae and (b) Rhabdoviridae families. The color gradient reflects the levels of identity, ranging from blue (low identity) to red (high identity), according to the lateral scale. (a) In Lispiviridae, we observed two main groupings: group 1, with the viruses CxLispV-SP_03, 09, 13, 15, and 12 and Canya virus, showing high nucleotide identity (> 80%), indicating a close evolutionary relationship. Group 2, made up of viruses such as Lisp_Pedras_lispivirus and Lisp_Canmo_PELV, with identity lower than 60% in relation to group 1, suggests a distinct lineage. (b) In Rhabdoviridae, four distinct groups were delimited: group 1, made up of CxRhabV-SP_05 and 16, and Stang virus, with identity > 80%; group 2, with viruses such as CxRhabV-SP_08, CxRhabV-SP_06, CxRhabV-SP_10, and Rhab_Delta_Stangha_ELSVV, with identity between ∼70 and 75%; group 3, including AnRhabV-SP_01, Sanxia Water Strider Virus 5, Hangzhou rhabdovirus 5, and representatives of the genus Alpharhabdovirus, with moderate identity (50%–65%); group 4, represented by more divergent viruses such as Merida virus, Rhab_Alph_Metra_MERV, and Rhab_Alph_Oshima_OHLVD, with less than 50% identity, showing greater phylogenetic distance. [file AV-2026-8104754-s002.docx]

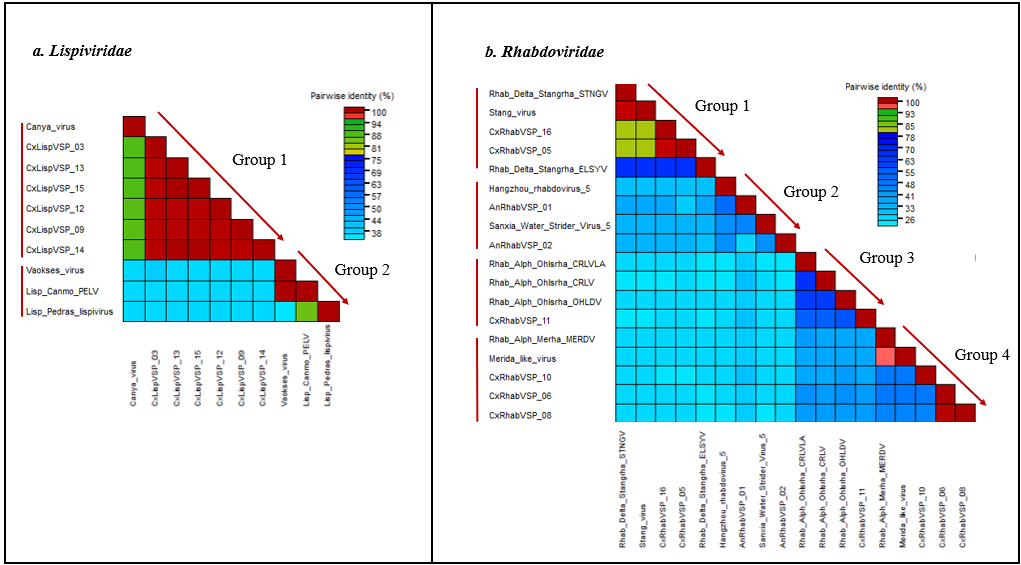
**Figure S3.**

**Figure S3. Identity analysis of the RdRp (L) protein between viruses from the *Lispiviridae* and *Rhabdoviridae* families. Identity matrix generated with the Sequence**. Demarcation Tool (SDT) software, representing the CxLispV-SP, CxRhabV-SP and CxRhabV-SP sequences of the L protein (RdRp) of viruses from the (a) *Lispiviridae* and (b) *Rhabdoviridae* families. The color gradient reflects the levels of identity, ranging from blue (low identity) to red (high identity), according to the lateral scale.(a) In *Lispiviridae*, we observed two main groupings: group 1, with the viruses *CxLispV-SP_03*, 09, 13, 15, 12 and *Canya virus*, showing high nucleotide identity (>80%), indicating a close evolutionary relationship. Group 2, made up of viruses such as *Lisp_Pedras_lispivirus* and *Lisp_Canmo_PELV*, with identity lower than 60% in relation to group 1, suggesting a distinct lineage. (b) In *Rhabdoviridae*, four distinct groups were delimited: group 1, made up of *CxRhabV-SP_05*, 16, and *Stang virus*, with identity >80%; group 2, with viruses such as *CxRhabV-SP_08*, *CxRhabV-SP_06*, *CxRhabV-SP_10* and *Rhab_Delta_Stangha_ELSVV*, with identity between ~70-75%; group 3, including *AnRhabV-SP_01*, Sanxia water strider virus 5, Hangzhou rhabdovirus 5 and representatives of the genus *Alpharhabdovirus*, with moderate identity (50-65%); group 4, represented by more divergent viruses such as Merida virus, *Rhab_Alph_Metra_MERV*, and *Rhab_Alph_Oshima_OHLVD*, with less than 50% identity, showing greater phylogenetic distance.
