# Supplementary material for: Putative Novel Viruses in the Families Lispiviridae and Rhabdoviridae Detected in Culex and Anopheles Mosquitoes Collected at the São Paulo Zoo
Source: Adv Virol. 2026 Jun 29;2026:8104754. doi: 10.1155/av/8104754 (PMC13315819; doi:10.1155/av/8104754)
Supplement: Supplementary file 4 — Supporting Information 4 Table S1: composition of metagenomic pools based on taxonomic and geospatial criteria. Specimens were collected from ground and canopy strata across eight sites at the FPZSP. [file AV-2026-8104754-s009.docx]

**Table S1 - Composition of metagenomic pools based on taxonomic and geospatial criteria. Specimens were collected from ground and canopy strata across eight sites at the FPZSP.**

| **Sample number** | **Species** | **Trap ID (ALL)** | **Sampling Site** | **Trap Height** | **Location Name** | **Coordinates** |  |
| --- | --- | --- | --- | --- | --- | --- | --- |
|  |  |  |  |  |  |  |  |
| **Meta 21** | *Anopheles (Nys.) strodei* | 22 | Canopy | 7,5 meters | Enclosure 113 (E113) | 23°38'58.3"S |  |
|  | *Anopheles (Nys.) strodei* |  | Ground | 1,5 meters |  | 46°37'03.9"W |  |
|  | *Anopheles (Nys.) strodei* |  | Canopy | 7,5 meters | Lake bridge (LB) | 23°39'08.1"S |  |
|  | *Anopheles (Nys.) strodei* |  | Ground | 1,5 meters |  | 46°37'03.9"W |  |
|  | *Anopheles (Nys.) strodei* |  | Canopy | 6 meters | Lake 70 (L70) | 23°39'04.0"S |  |
|  | *Anopheles (Nys.) strodei* |  | Ground | 1,5 meters |  | 46°37'11.9"W |  |
|  | *Anopheles (Nys.) strodei* |  | Canopy | 10 meters | Corridor 61 (C61) | 23°39'01.5"S |  |
|  | *Anopheles (Nys.) strodei* |  | Ground | 1,5 meters |  | 46°37'03.0"W |  |
|  | *Anopheles (Nys.) strodei* |  | Canopy | 8 meters | Recinto 69 (R69) | 23°39'11.2"S |  |
|  | *Anopheles (Nys.) strodei* |  | Ground | 1,5 meters |  | 46°36'59.7"W |  |
|  | *Anopheles (Nys.) strodei* |  | Canopy | 6 meters | Flamingos enclosure (FE) | 23°38'55.2"S |  |
|  | *Anopheles (Nys.) strodei* |  | Ground | 1,5 meters |  | 46°37'16.8"W |  |
|  | *Anopheles (Nys.) strodei* |  | Canopy | 8 meters | Bird Forest (BF) | 23°38'54.0"S |  |
|  | *Anopheles (Nys.) strodei* |  | Ground | 1,5 meters |  | 46°37'13.4"W |  |
|  | *Anopheles (Nys.) strodei* |  | Canopy | 8 meters | Extra (EX) | 23°38'48.2"S |  |
|  | *Anopheles (Nys.) strodei* |  | Ground | 1,5 meters |  | 46°37'14.4"W |  |
| **Meta 22** | *Culex (Cux.) chidesteri* | 50 | Canopy | 8 meters | Extra (EX) | 23°38'48.2"S |  |
|  |  |  | Ground | 1,5 meters |  | 46°37'14.4"W |  |
| **Meta 23** | *Culex (Cux.) chidesteri* | 50 | Canopy | 8 meters | Extra (EX) | 23°38'48.2"S |  |
|  |  |  | Ground | 1,5 meters |  | 46°37'14.4"W |  |
| **Meta 28** | *Culex (Cux.) renatoi* | 21 | Canopy | 6 meters | Flamingos enclosure (FE) | 23°38'55.2"S |  |
|  |  |  | Ground | 1,5 meters |  | 46°37'16.8"W |  |
| **Meta 41** | *Culex (Cux.) chidesteri* | 28 | Canopy | 8 meters | Bird Forest (BF) | 23°38'54.0"S |  |
|  |  |  | Ground | 1,5 meters |  | 46°37'13.4"W |  |
| **Meta 43** | *Culex (Cux.) chidesteri* | 35 | Canopy | 10 meters | Corridor 61 (C61) | 23°39'01.5"S |  |
|  |  |  | Ground | 1,5 meters |  | 46°37'03.0"W |  |
| **Meta 46** | *Culex (Cux.) chidesteri* | 26 | Canopy | 6 meters | Recinto 69 (R69) | 23°39'11.2"S |  |
|  |  |  | Ground | 1,5 meters |  | 46°36'59.7"W |  |
| **Meta 47** | *Culex (Cux.) chidesteri* | 33 | Canopy | 8 meters | Recinto 69 (R69) | 23°39'11.2"S |  |
|  |  |  | Ground | 1,5 meters |  | 46°36'59.7"W |  |
