# Supplementary material for: Putative Novel Viruses in the Families Lispiviridae and Rhabdoviridae Detected in Culex and Anopheles Mosquitoes Collected at the São Paulo Zoo
Source: Adv Virol. 2026 Jun 29;2026:8104754. doi: 10.1155/av/8104754 (PMC13315819; doi:10.1155/av/8104754)
Supplement: Supplementary file 5 — Supporting Information 5 Table S2: sequencing and assembly metrics of metagenomically assembled viral genomes identified in mosquito samples. [file AV-2026-8104754-s004.docx]

**Table S2 - Sequencing and assembly metrics of metagenomically assembled viral genomes identified in mosquito samples.**

| **Samplename** | **GenBank Accession** | **BioSample Accession** | **SRA Accession** | **Assembled Contigs (Size)** | **Sample Name** | **Assembled Contigs** | **Mapped Reads** | **Avg. Depth** | **Breadth (%)** |
| --- | --- | --- | --- | --- | --- | --- | --- | --- | --- |
| *CxLispV-SP_03* | PX833269 | SAMN55864756 | SRR37305201 | 6149 bp | Mosq_lib22 | 1.606 | 328 | 12.3627 | 100 |
| *CxLispV-SP_09* | PX833270 | SAMN55864757 | SRR37305200 | 6676 bp | Mosq_lib23 | 405 | 10329 | 201.52 | 99 |
| *CxLispV-SP_12* | PX833271 | SAMN55864759 | SRR37305198 | 13713 bp | Mosq_lib41 | 209 | 3534 | 58.3115 | 100 |
| *CxLispV-SP_13* | PX833272 | SAMN55864760 | SRR37305197 | 9433 bp | Mosq_lib43 | 271 | 1307 | 31.0861 | 99 |
| *CxLispV-SP_14* | PX833273 | SAMN55864761 | SRR37305196 | 6573 bp | Mosq_lib46 | 1.772 | 735 | 24.7047 | 99 |
| *CxLispV-SP_15* | PX833274 | SAMN55864762 | SRR37305195 | 6562 bp | Mosq_lib47 | 197 | 470 | 15.0296 | 100 |
| *AnRhabV-SP_01* | PX833262 | SAMN55864755 | SRR37305202 | 7666 bp | Mosq_lib21 | 212 | 425 | 12.6554 | 100 |
| *AnRhabV-SP_02* | PX833263 | SAMN55864755 | SRR37305202 | 5140 bp | Mosq_lib21 | 212 | 240 | 10.7623 | 100 |
| *Culex-SP_04* | PX833264 | SAMN55864756 | SRR37305201 | 5422 bp | Mosq_lib22 | 1.606 | 1337 | 55.2387 | 97 |
| *CxRhabV-SP_05* | PX833265 | SAMN55864756 | SRR37305201 | 8263 bp | Mosq_lib22 | 1.606 | 406 | 10.851 | 99 |
| *CxRhabV-SP_06* | PX833266 | SAMN55864756 | SRR37305201 | 6016 bp | Mosq_lib22 | 1.606 | 1255 | 45.621 | 100 |
| *CxRhabV-SP_08* | PX833267 | SAMN55864757 | SRR37305200 | 11184 bp | Mosq_lib23 | 405 | 324 | 85.0622 | 100 |
| *CxRhabV-SP_10* | PX833260 | SAMN55864758 | SRR37305199 | 10132 bp | Mosq_lib28 | 5.011 | 824 | 28.3604 | 100 |
| *CxRhabV-SP_11* | PX833261 | SAMN55864758 | SRR37305199 | 11373 bp | Mosq_lib28 | 5.011 | 9154 | 205.81 | 100 |
| CuRhabV-SP_16 | PX833268 | SAMN55864762 | SRR37305195 | 7138 bp | Mosq_lib47 | 197 | 376 | 11.226 | 100 |

Summary of accession numbers, BioSample identifiers, sequencing runs, assembled contig sizes, sample origin, number of assembled contigs, mapped reads, average sequencing depth, and genome breadth of coverage (%) for each viral genome recovered from mosquito libraries. Genome sizes range from ~5 kb to ~13 kb. Breadth of coverage values (97–100%) support the robustness of most assemblies, although completeness status varies according to genome recovery.
