# Supplementary material for: Putative Novel Viruses in the Families Lispiviridae and Rhabdoviridae Detected in Culex and Anopheles Mosquitoes Collected at the São Paulo Zoo
Source: Adv Virol. 2026 Jun 29;2026:8104754. doi: 10.1155/av/8104754 (PMC13315819; doi:10.1155/av/8104754)
Supplement: Supplementary file 6 — Supporting Information 6 Table S3: similarity analysis of viral genomes identified in mosquito samples using BLASTp. [file AV-2026-8104754-s005.docx]

**Table S3 -** S**imilarity Analysis of Viral Genomes Identified in Mosquito Samples Using BLASTp.**

| **Samplename** | **Size in base pairs** | **Best-hit GenbankID** | **Coverage** | **E-value** | **Identity** | **Family** |
| --- | --- | --- | --- | --- | --- | --- |
| *CxLispV-SP_03* | 6149 | QRW41713.1 | 97% | 0× 10^0^ | 87.75% | *Lispiviridae* |
| *CxLispV-SP_09* | 6676 | QRW41713.1 | 94% | 0× 10^0^ | 87.52% | *Lispiviridae* |
| *CxLispV-SP_12* | 13713 | QRW41713.1 | 45% | 0× 10^0^ | 87.52% | *Lispiviridae* |
| *CxLispV-SP_13* | 9433 | QRW41713.1 | 66% | 0× 10^0^ | 87.57% | *Lispiviridae* |
| *CxLispV-SP_14* | 6573 | QRW41713.1 | 95% | 0× 10^0^ | 87.57% | *Lispiviridae* |
| *CxLispV-SP_15* | 6562 | QRW41713.1 | 95% | 0× 10^0^ | 87.57% | *Lispiviridae* |
| *AnRhabV-SP_01* | 7666 | UHK03252.1 | 22% | 4 × 10^-157^ | 50.43% | *Rhabdoviridae* |
| *AnRhabV-SP_02* | 5140 | YP_009289352.1 | 95% | 0× 10^0^ | 41.69% | *Rhabdoviridae* |
| *Culex-SP_04* | 5422 | UUG74169.1 | 27% | 6 × 10^-126^ | 45.23% | *Rhabdoviridae* |
| *CxRhabV-SP_05* | 8263 | QRW41829.1 | 82% | 0× 10^0^ | 82.13% | *Rhabdoviridae* |
| *CxRhabV-SP_06* | 6016 | WPK42782.1 | 93% | 0× 10^0^ | 47.96% | *Rhabdoviridae* |
| *CxRhabV-SP_08* | 11184 | WPK42782.1 | 55% | 0× 10^0^ | 46.37% | *Rhabdoviridae* |
| *CxRhabV-SP_10* | 10132 | WPK42782.1 | 61% | 0× 10^0^ | 50.14% | *Rhabdoviridae* |
| *CxRhabV-SP_11* | 11373 | UYE93948.1 | 55% | 0× 10^0^ | 56.55% | *Rhabdoviridae* |
| CuRhabV-SP_16 | 7138 | QRW41834.1 | 95% | 0× 10^0^ | 81.34% | *Rhabdoviridae* |
