# Supplementary material for: Putative Novel Viruses in the Families Lispiviridae and Rhabdoviridae Detected in Culex and Anopheles Mosquitoes Collected at the São Paulo Zoo
Source: Adv Virol. 2026 Jun 29;2026:8104754. doi: 10.1155/av/8104754 (PMC13315819; doi:10.1155/av/8104754)
Supplement: Supplementary file 7 — Supporting Information 7 Table S4: comparative analysis of the genomes and ORF structures of CxLispV and AnRhabV. [file AV-2026-8104754-s006.docx]

**Table S4 - Comparative Analysis of the Genomes and ORF Structures of CxLispV and AnRhabV.**

| **Samplename** | **Genome size (bp)** | **Protein** | **Length (aa)** | **ORF position (nt)** | **Accession (Blastp/CDD)** | **E-value (Blastp)** | **E-value (CDD)** |
| --- | --- | --- | --- | --- | --- | --- | --- |
| *CxLispV-SP_03* | 6149 | RNA-dependent RNA polymerase (RdRp) | 1968 | 0050 - 5956 | QRW41710.1 / cl15638 | 1< 10⁻¹⁸⁰ | 4.75 × 10^-102^ |
| *CxLispV-SP_09* | 6676 | RNA-dependent RNA polymerase (RdRp) | 2099 | 0091 - 6390 | QRW41710.1 / cl15638 | 1< 10⁻¹⁸⁰ | 4.61 × 10^-102^ |
| *CxLispV-SP_12* | 13713 | Hypothetical protein (HP) | 471 | 0006 - 1421 | KXJ70270.1 / – | 7 × 10^-110^ | – |
|  |  | Hypothetical protein (HP) | 583 | 1556 - 3307 | QRW41711.1 / – | – | – |
|  |  | Glycoprotein (G) | 566 | 4531 - 6231 | QRW41711.1 / – | 1< 10⁻¹⁸⁰ | – |
|  |  | Hypothetical protein (HP) | 119 | 6422 - 6781 | QRW41709.1 / – | 7 × 10^-155^ | – |
|  |  | RNA-dependent RNA polymerase (RdRp) | 2099 | 7296 - 13595 | QRW41710.1 / cl15638 | 1< 10⁻¹⁸⁰ | 2.08 × 10^-101^ |
| *CxLispV-SP_13* | 9433 | Glycoprotein (G) | 566 | 0104 - 1804 | QRW41711.1 / – | 1< 10⁻¹⁸⁰ | – |
|  |  | RNA-dependent RNA polymerase (RdRp) | 2099 | 2873 - 9172 | QRW41710.1 / cl15638 | 1< 10⁻¹⁸⁰ | 2.86 × 10^-113^ |
| *CxLispV-SP_14* | 6573 | RNA-dependent RNA polymerase (RdRp) | 2099 | 0082 - 6381 | QRW41710.1 / cl15638 | 1< 10⁻¹⁸⁰ | 1.20 × 10^-102^ |
| *CxLispV-SP_15* | 6562 | RNA-dependent RNA polymerase (RdRp) | 2099 | 0071 - 6370 | QRW41710.1 / cl15638 | 1< 10⁻¹⁸⁰ | 2.86 × 10^-103^ |
| *AnRhabV-SP_01* | 7666 | Nucleoprotein (N) | 376 | 0167 - 1297 | XXL23990.1 / cl03939 | 6 × 10^-54^ | 1.91 × 10^-58^ |
|  |  | Hypothetical protein (HP) | 336 | 1395 - 2405 | – | – | – |
|  |  | Hypothetical protein (HP) | 221 | 2511 - 3176 | – | – | – |
|  |  | Glycoprotein (G) | 637 | 3641 - 5554 | YP_009289351.1 / – | 9× 10^-76^ | – |
|  |  | RNA-dependent RNA polymerase (RdRp) | 590 | 5892 - 7664 | UHK03252.1 / cl15638 | 1< 10⁻¹⁸⁰ | 3.25 × 10^-4^ |
| *AnRhabV-SP_02* | 5140 | RNA-dependent RNA polymerase (RdRp) | 1705 | 0010 - 5127 | YP_009289352.1 / cl15638 | 1< 10⁻¹⁸⁰ | 3.40 × 10^-63^ |

The symbol ( **-** ) indicates predicted ORFs with no detectable significant similarity in Blastp and CDD analyses.

***Supplementary Table 5 (continued).***

| **Samplename** | **Genome size (bp)** | **Protein** | **Length (aa)** | **ORF position (nt)** | **Accession (Blastp/CDD)** | **E-value (Blastp)** | **E-value (CDD)** |
| --- | --- | --- | --- | --- | --- | --- | --- |
| *Culex-SP_04* | 5422 | Nucleoprotein (N) | 446 | 0195 - 1534 | EAT48846.1 / cl20153 | 8 × 10^-83^ | 1.47 × 10^-44^ |
|  |  | Hypothetical protein (HP) | 302 | 1618 - 2526 | – | – | – |
|  |  | Hypothetical protein (HP) | 182 | 2586 - 3134 | – | – | – |
|  |  | Glycoprotein (G) | 507 | 3170 - 4693 | AWJ96717.1 | 8 × 10^-140^ | – |
| *CxRhabV-SP_05* | 8263 | Glycoprotein (G) | 233 | 0194 - 895 | QRW41833.1/ cl03047 | 4 × 10^-91^ | 7.94 × 10^-61^ |
|  |  | RNA-dependent RNA polymerase (RdRp) | 442 | 1320 - 2648 | QRW41829.1 / cl15638 | 1< 10⁻¹⁸⁰ | – |
|  |  | RNA-dependent RNA polymerase (RdRp) | 1711 | 3043 - 8178 | QRW41829.1 / cl15638 | 1< 10⁻¹⁸⁰ | 5.88 × 10^-20^ |
| *CxRhabV-SP_06* | 6016 | RNA-dependent RNA polymerase (RdRp) | 1880 | 0035 - 5677 | XQP09949.1 / cl15638 | 1< 10⁻¹⁸⁰ | 1< 10⁻¹⁸⁰ |
| *CxRhabV-SP_08* | 11184 | Nucleoprotein (N) | 446 | 0113 - 1453 | EAT48846.1 / cl20153 | 8 × 10^-83^ | 1.68 × 10^-45^ |
|  |  | Hypothetical protein (HP) | 302 | 1537 - 2445 | – | – | – |
|  |  | Hypothetical protein (HP) | 182 | 2505 - 3053 | – | – | – |
|  |  | Glycoprotein (G) | 507 | 3089 - 4612 | UUG74169.1/ cl03047 | 3 × 10^-140^ | 1.99 × 10^-24^ |
|  |  | RNA-dependent RNA polymerase (RdRp) | 2120 | 4709 - 11071 | QRW41829.1 / cl15638 | 1< 10⁻¹⁸⁰ | 1< 10⁻¹⁸⁰ |
| *CxRhabV-SP_10* | 10132 | Nucleoprotein (N) | 470 | 0125 - 1537 | XP_055543613.1 / cl20153 | 2 × 10^-74^ | 5.11 × 10^-59^ |
|  |  | Hypothetical protein (HP) | 352 | 1637 - 2695 | – | – | – |
|  |  | Hypothetical protein (HP) | 173 | 2730 - 3251 | – | – | – |
|  |  | RNA-dependent RNA polymerase (RdRp) | 2136 | 3496 - 9906 | AWJ96718.1 / cl15638 | 1< 10⁻¹⁸⁰ | 1< 10⁻¹⁸⁰ |
| *CxRhabV-SP_11* | 11373 | Nucleoprotein (N) | 434 | 236 - 1540 | YP_009388612.1/ cl20153 | 8 × 10^-94^ | 2 × 10^-57^ |
|  |  | Hypothetical protein (HP) | 361 | 1617 - 2702 | – | – | – |
|  |  | Matrix protein (M) | 173 | 2747 - 3267 | YP_010086784.1 / – | 2 × 10^-28^ | – |
|  |  | Glycoprotein (G) | 506 | 3369 - 4889 | YP_009289351.1/ cl03047 | 2 × 10^-157^ | 3.9 × 10^-39^ |
|  |  | RNA-dependent RNA polymerase (RdRp) | 2129 | 4935 - 11324 | YP_010086786.1 / cl15638 | 1< 10⁻¹⁸⁰ | 1< 10⁻¹⁸⁰ |
| *CuRhabV-SP_16* | 7138 | RNA-dependent RNA polymerase (RdRp) | 2285 | 179 - 7036 | QRW41829.1 /cl15638 | 1< 10⁻¹⁸⁰ | 2.9 × 10^-19^ |

The symbol ( **-** ) indicates predicted ORFs with no detectable significant similarity in Blastp and CDD analyses. E-value reported as 0.0 were below the computational precision limit of BLAST and are herein represented as < 1 × 10⁻¹⁸⁰ for visualization purposes.
