# Supplementary material for: Putative Novel Viruses in the Families Lispiviridae and Rhabdoviridae Detected in Culex and Anopheles Mosquitoes Collected at the São Paulo Zoo
Source: Adv Virol. 2026 Jun 29;2026:8104754. doi: 10.1155/av/8104754 (PMC13315819; doi:10.1155/av/8104754)
Supplement: Supplementary file 8 — Supporting Information 8 Table S5: Pfam‐predicted functional domains, genomic positions, and statistical significance (i‐Evalue) identified in RdRp (L protein) sequences from mosquito‐associated viruses. [file AV-2026-8104754-s007.docx]

**Table S5 - Pfam‑predicted functional domains, genomic positions, and statistical significance (i‑Evalue) identified in RdRp (L protein) sequences from mosquito‑associated viruses.**

| **Samplename** | **RdRp_Size** | **Pfam ID** | **Pfam ID** | **Position** | **i-Evalue** |
| --- | --- | --- | --- | --- | --- |
|  | **(Base pairs)** |  |  |  |  |
| *CxLispV-SP_03* | 1968 | PF00946 | Mononeg_RNA_pol | 73 – 932 | 8.7 × 10^-132^ |
|  |  | PF14318 | Mononeg_mRNAcap | 974 – 1163 | 5.4 × 10^-28^ |
|  |  | PF01728 | FtsJ | 1640 – 1753 | 1.1 × 10^-1^ |
| *CxLispV-SP_09* | 2099 | PF00946 | Mononeg_RNA_pol | 198 – 1063 | 8.2 × 10^-133^ |
|  |  | PF14318 | Mononeg_mRNAcap | 1105 – 1294 | 5.9 × 10^-28^ |
|  |  | PF01728 | FtsJ | 1771 – 1884 | 1.1 × 10^-2^ |
| *CxLispV-SP_12* | 2099 | PF00946 | Mononeg_RNA_pol | 198 – 1063 | 2.2 × 10^-133^ |
|  |  | PF14318 | Mononeg_mRNAcap | 1105 – 1294 | 5.9 × 10^-28^ |
|  |  | PF01728 | FtsJ | 1771 – 1884 | 1.1 × 10^-2^ |
| *CxLispV-SP_13* | 2099 | PF00946 | Mononeg_RNA_pol | 239 – 1085 | 6.9 × 10^-212^ |
|  |  | PF14314 | Methyltrans_Mon_2nd | 1653 – 1914 | 3.4 × 10^-57^ |
|  |  | PF14318 | Mononeg_mRNAcap | 1098 – 1327 | 3.9 × 10^-48^ |
|  |  | PF21080 | Methyltrans_Mon_1st | 1377 – 1571 | 2.4 × 10^-28^ |
|  |  | PF21081 | Methyltrans_Mon_3rd | 1918 – 2025 | 1.6 × 10^-5^ |
|  |  | PF03640 | Lipoprotein_15 | 437 – 454 | 1.2 × 10^-1^ |
|  |  | PF01728 | FtsJ | 1767 – 1861 | 2.8 × 10^-1^ |
| *CxLispV-SP_14* | 2099 | PF00946 | Mononeg_RNA_pol | 1 – 837 | 2.2 × 10^-213^ |
|  |  | PF14314 | Methyltrans_Mon_2nd | 1406 – 1669 | 6.2 × 10^-57^ |
|  |  | PF14318 | Mononeg_mRNAcap | 853 – 1082 | 3.1 × 10^-50^ |
|  |  | PF21080 | Methyltrans_Mon_1st | 1132 – 1324 | 9.2 × 10^-35^ |
|  |  | PF21081 | Methyltrans_Mon_3rd | 1735 – 1792 | 2.7 × 10^-5^ |
|  |  | PF01728 | FtsJ | 1428 – 1619 | 2.1 × 10^-1^ |
| *CxLispV-SP_15* | 2099 | PF00946 | Mononeg_RNA_pol | 197 – 1063 | 2.6 × 10^-133^ |
|  |  | PF14318 | Mononeg_mRNAcap | 1105 – 1294 | 5.9 × 10^-28^ |
|  |  | PF01728 | FtsJ | 1771 – 1884 | 1.1 × 10^-2^ |

***Supplementary Table 3 (continued).***

| **Samplename** | **RdRp_Size** | **Pfam ID** | **Pfam ID** | **Position** | **i-Evalue** |
| --- | --- | --- | --- | --- | --- |
|  | **(Base pairs)** |  |  |  |  |
| *AnRhabV-SP_01* | 591 | PF00946 | Mononeg_RNA_pol | 189 – 585 | 2.6 × 10^-76^ |
|  |  | PF00657 | Lipase_GDSL | 252 – 290 | 3.1 × 10^-1^ |
| *AnRhabV-SP_02* | 1705 | PF00946 | Mononeg_RNA_pol | 1 – 469 | 2.4 × 10^-86^ |
|  |  | PF14318 | Mononeg_mRNAcap | 518 – 761 | 2.6 × 10^-24^ |
|  |  | PF14314 | Methyltrans_Mon_2nd | 1183 – 1371 | 3.2 × 10^-13^ |
|  |  | PF03902 | Gal4_dimer | 118 – 144 | 7.0 × 10^-1^ |
| *Culex-SP_04* | – | – | – | – | – |
| *CxRhabV-SP_05* | 1711 | PF00946 | Mononeg_RNA_pol | 1 – 517 | 1.6 × 10^-79^ |
|  |  | PF14318 | Mononeg_mRNAcap | 571 – 816 | 3 × 10^-35^ |
|  |  | PF14314 | Methyltrans_Mon_2nd | 1211 – 1415 | 6.3 × 10^-12^ |
|  | 442 | PF00946 | Mononeg_RNA_pol | 236 – 437 | 4.2 × 10^-25^ |
| *CxRhabV-SP_06* | 1880 | PF00946 | Mononeg_RNA_pol | 1 – 837 | 2 × 10^-213^ |
|  |  | PF14314 | Methyltrans_Mon_2nd | 1406 – 1669 | 5.6 × 10^-57^ |
|  |  | PF14318 | Mononeg_mRNAcap | 853 – 1082 | 2.8 × 10^-50^ |
|  |  | PF21080 | Methyltrans_Mon_1st | 1132 – 1324 | 8.4 × 10^-35^ |
|  |  | PF21081 | Methyltrans_Mon_3rd | 1735 – 1792 | 2.5 × 10^-5^ |
|  |  | PF01728 | FtsJ | 1428 – 1619 | 1.9 × 10^-4^ |
| *CxRhabV-SP_08* | 2120 | PF00946 | Mononeg_RNA_pol | 175 – 1077 | 2 × 10^-213^ |
|  |  | PF14314 | Methyltrans_Mon_2nd | 1646 – 1909 | 2.7 × 10^-56^ |
|  |  | PF14318 | Mononeg_mRNAcap | 1093 – 1322 | 3.3 × 10^-50^ |
|  |  | PF21080 | Methyltrans_Mon_1st | 1372 – 1564 | 9.8 × 10^-35^ |
|  |  | PF21081 | Methyltrans_Mon_3rd | 1975 – 2032 | 2.8 × 10^-5^ |
|  |  | PF01728 | FtsJ | 1668 – 1859 | 8.9 × 10^-4^ |
| *CxRhabV-SP_10* | 2136 | PF00946 | Mononeg_RNA_pol | 239 – 1085 | 6.3 × 10^-212^ |
|  |  | PF14314 | Methyltrans_Mon_2nd | 1653 – 1914 | 3.1 × 10^-57^ |
|  |  | PF14318 | Mononeg_mRNAcap | 1098 – 1327 | 3.6 × 10^-48^ |
|  |  | PF21080 | Methyltrans_Mon_1st | 1377 – 1571 | 2.2 × 10^-28^ |
|  |  | PF21081 | Methyltrans_Mon_3rd | 1918 – 2025 | 1.4 × 10^-5^ |
|  |  | PF03640 | Lipoprotein_15 | 437 – 454 | 1.1 × 10^-1^ |
|  |  | PF01728 | FtsJ | 1767 – 1861 | 2.6 × 10^-1^ |

***Supplementary Table 3 (continued).***

| **Samplename** | **RdRp_Size** | **Pfam ID** | **Pfam ID** | **Position** | **i-Evalue** |
| --- | --- | --- | --- | --- | --- |
|  | **(Base pairs)** |  |  |  |  |
| *CxRhabV-SP_11* | 2128 | PF00946 | Mononeg_RNA_pol | 58 – 1089 | 8.7 × 10^-233^ |
|  |  | PF14318 | Mononeg_mRNAcap | 1102 – 1331 | 2.1 × 10^-54^ |
|  |  | PF14314 | Methyltrans_Mon_2nd | 1659 – 1919 | 5.1 × 10^-54^ |
|  |  | PF21080 | Methyltrans_Mon_1st | 1381 – 1554 | 3.3 × 10^-28^ |
|  |  | PF21081 | Methyltrans_Mon_3rd | 1986 – 2034 | 3.4 × 10^-5^ |
| CuRhabV-SP_16 | 2285 | PF00946 | Mononeg_RNA_pol | 58 – 1089 | 4.7 × 10^-146^ |
|  |  | PF14318 | Mononeg_mRNAcap | 1102 – 1331 | 4.5 × 10^-35^ |
|  |  | PF14314 | Methyltrans_Mon_2nd | 1659 – 1919 | 5.2 × 10^-12^ |

**Abbreviations:** Mononeg_RNA_pol, *Mononegavirales* RNA dependent RNA polymerase; Mononeg_mRNAcap, *Mononegavirales* mRNA-capping region V; FtsJ, FtsJ-like methyltransferase; Methyltrans_Mon_2nd, Virus-capping methyltransferase, MT domain; Methyltrans_Mon_1st, Virus-capping methyltransferase, connector domain; ethyltrans_Mon_3rd, Virus-capping methyltransferase, C-terminal; Lipase_GDSL, GDSL-like Lipase/Acylhydrolase; Gal4_dimer, Gal4-like dimerisation domain; and Lipoprotein_15, Secreted repeat of unknown .
