# Supplementary material for: Putative Novel Viruses in the Families Lispiviridae and Rhabdoviridae Detected in Culex and Anopheles Mosquitoes Collected at the São Paulo Zoo
Source: Adv Virol. 2026 Jun 29;2026:8104754. doi: 10.1155/av/8104754 (PMC13315819; doi:10.1155/av/8104754)
Supplement: Supplementary file 9 — Supporting Information 9 Table S6: distribution and conservation of functional motifs in RdRp. [file AV-2026-8104754-s003.docx]

**Table S6.**

**Distribution and conservation of functional motifs in RdRp. presents quantitative data on the relative frequency of amino acids in specific positions of motifs A, B and C, allowing a detailed analysis of the conservation and variation patterns between residues, which contributes to the identification of functionally relevant regions in the RdRp protein.**

| **Frequency** | | | | | | | | | | | | | | | | | | | | | | | | | | | | |
| --- | --- | --- | --- | --- | --- | --- | --- | --- | --- | --- | --- | --- | --- | --- | --- | --- | --- | --- | --- | --- | --- | --- | --- | --- | --- | --- | --- | --- |
| **Motif** | **Position / Percentage** | | | | | | | | | | | | | | | | | | | | | | | | | | | |
|  | **1** |  | **2** |  | **3** |  | **4** |  | **5** |  | **6** |  | **7** |  | **8** |  | **9** |  | **10** |  | **11** |  | **12** |  | **13** |  | **14** |  |
| Motif A | N | 34.6% | H | 34.6% | I | 38.5% | D | 100% | F | 65.4% | K | 30.8% | K | 69.2% | W | 100% | N | 100% | N | 34.6% | H | 57.7% | N | 38.5% |  |  |  |  |
|  | V | 38.8% | G | 43.6% | V | 30.8% |  |  |  |  | V | 19.2% |  |  |  |  |  |  | I | 34.6% | N | 19.2% | Q | 34.6% |  |  |  |  |
|  | T | 23.1% | N | 26.9% | M | 23.1% |  |  | Y | 34.6% | S | 19.2% | S | 30.8% |  |  |  |  | S | 19.2% | F | 15.4% | M | 26.9% |  |  |  |  |
|  | I | 7.7% | S | 3.8% | L | 7.7% |  |  |  |  | E | 14.4% |  |  |  |  |  |  | T | 3.8% | Y | 7.7% |  |  |  |  |  |  |
| Motif B | E | 100% | G | 100% | L | 65.4% | R | 61.5% | Q | 100% | K | 100% | G | 61.5% | W | 100% | T | 65.4% | L | 50% | I | 38.5% | T | 65.4% | I | 38.5% | G | 34.6% |
|  |  |  |  |  |  |  | M | 34.6% |  |  |  |  | M | 26.9% |  |  |  |  | V | 23.1% | F | 23.1% |  |  | V | 26.9% | L | 34.6% |
|  |  |  |  |  | I | 34.6% | C | 3.8% |  |  |  |  | L | 11.5% |  |  | S | 34.6% | I | 19.2% | V | 15.4% | N | 34.6% | Y | 15.4% | S | 19.2% |
|  |  |  |  |  |  |  |  |  |  |  |  |  |  |  |  |  |  |  | W | 7.7% | L | 12% |  |  | L | 15.4% | V | 7.7% |
| Motif C | G | 65.4% | Q | 100% | G | 100% | D | 100% | N | 100% | Q | 100% | V | 88.5% | L | 42.3% |  |  |  |  |  |  |  |  |  |  |  |  |
|  |  |  |  |  |  |  |  |  |  |  |  |  |  |  | C | 38.5% |  |  |  |  |  |  |  |  |  |  |  |  |
|  | A | 34.6% |  |  |  |  |  |  |  |  |  |  | I | 34.6% | I | 14.4% |  |  |  |  |  |  |  |  |  |  |  |  |
|  |  |  |  |  |  |  |  |  |  |  |  |  |  |  | V | 3.8% |  |  |  |  |  |  |  |  |  |  |  |  |

numbered sequentially (1–14) according to the alignment of each motif. Percentage values indicate the frequency of occurrence of each amino acid residue at a given position across the analyzed Sequence.
